# Disulfiram downregulates ferredoxin 1 to maintain copper homeostasis and inhibit inflammation in cerebral ischemia/reperfusion injury

**DOI:** 10.1038/s41598-024-64981-x

**Published:** 2024-07-02

**Authors:** Shuai Yang, Xudong Li, Jinhong Yan, Fangchao Jiang, Xuehui Fan, Jing Jin, Weihua Zhang, Di Zhong, Guozhong Li

**Affiliations:** 1 The First Afliated Hospital of Harbin Medical University, 23 You Zheng Street, Harbin, 150001 China; 2 The Fourth Affiliated Hospital of Harbin Medical University, 37 Yiyuan Street, Harbin, 150001 China; 3 Heilongjiang Provincial Hospital, Harbin, China

**Keywords:** Cerebrovascular disorders, Hypoxic-ischaemic encephalopathy, Stroke

## Abstract

In the current study, we aimed to investigate whether disulfiram (DSF) exerts a neuroprotective role in cerebral ischemiareperfusion (CI-RI) injury by modulating ferredoxin 1 (FDX1) to regulate copper ion (Cu) levels and inhibiting inflammatory responses. To simulate CI-RI, a transient middle cerebral artery occlusion (tMCAO) model in C57/BL6 mice was employed. Mice were administered with or without DSF before and after tMCAO. Changes in infarct volume after tMCAO were observed using TTC staining. Nissl staining and hematoxylin–eosin (he) staining were used to observe the morphological changes of nerve cells at the microscopic level. The inhibitory effect of DSF on initial inflammation was verified by TUNEL assay, apoptosis-related protein detection and iron concentration detection. FDX1 is the main regulatory protein of copper death, and the occurrence of copper death will lead to the increase of HSP70 stress and inflammatory response. Cuproptosis-related proteins and downstream inflammatory factors were detected by western blotting, immunofluorescence staining, and immunohistochemistry. The content of copper ions was detected using a specific kit, while electron microscopy was employed to examine mitochondrial changes. We found that DSF reduced the cerebral infarction volume, regulated the expression of cuproptosis-related proteins, and modulated copper content through down regulation of FDX1 expression. Moreover, DSF inhibited the HSP70/TLR-4/NLRP3 signaling pathway. Collectively, DSF could regulate Cu homeostasis by inhibiting FDX1, acting on the HSP70/TLR4/NLRP3 pathway to alleviate CI/RI. Accordingly, DSF could mitigate inflammatory responses and safeguard mitochondrial integrity, yielding novel therapeutic targets and mechanisms for the clinical management of ischemia–reperfusion injury.

## Introduction

The incidence and mortality rates of cerebral ischemic disease are gradually increasing worldwide. Moreover, cerebral ischemic disease is the most common cause of permanent disability in adults^1^. Thrombolysis/thrombolysis is the most effective treatment for ischemic stroke. Since 2015, substantial advancements have been made in research exploring the treatment of acute ischemic stroke, leading to an expanded time window for emergency endovascular intervention, from the initial 6 h to a more extended period of 24 h^2^. Following cerebral ischemia, reperfusion of blood flow induces a cascade of injurious effects, commonly referred to as cerebral ischemia/reperfusion injury (CI/RI). This process involves various types of regulated cell death (RCD) and neuroinflammatory responses, considerably contributing to its progression^3,4^. Mitigating the adverse effects of reperfusion has been a persistent focal point for clinicians striving for breakthroughs.

In living organisms, cells exposed to extreme physicochemical or mechanical stressors may undergo an immediate and uncontrolled structural collapse, a phenomenon known as accidental cell death. Conversely, RCD involves specific signaling cascades and molecular-defined effector mechanisms, encompassing fundamental processes, such as organogenesis and tissue remodeling, eliminating unnecessary structures or cells, and controlling cell numbers. Additionally, RCD can be triggered by exogenous perturbations in the intracellular or extracellular microenvironments^5,6^. Currently, more than 10 types of RCDs have been identified, encompassing a diverse range of non-apoptotic RCD modalities, such as autophagy, pyroptosis, ferroptosis, and endogenous cell demise, in addition to the conventional apoptotic pathway^7,8^. In contrast to apoptosis, these RCD pathways elicit inflammatory responses within the body and have been increasingly associated with cancer^9^

CI/RI induces a robust inflammatory response initiated by damage-associated molecular patterns (DAMPs) released from injured cells, including signaling molecules, such as adenosine, heat shock proteins (HSPs), high-mobility group protein B1 (HMGB1), and interleukin (IL)^10–12^. In a healthy central nervous system, various types of DAMPs are expressed and released following injury to activate inflammatory signaling pathways^11^. The aforementioned DAMPs use pattern recognition receptors (PRRs) to initiate and augment immune responses^13^. Extracellular HSP70 is a typical DAMP^14^ PRRs are predominantly expressed in astrocytes and microglia, enabling the detection of pathogen-derived or endogenous ligand release. Toll-like receptors (TLRs) of the PRR family are an example of this phenomenon^15^. TLR4 plays a pivotal role and serves as the primary receptor for HMGB1^16^. In addition, HSP70 regulates TLR4^17,18^ TLR4 is most commonly associated with the expression and release of IL-1 and tumor necrosis factor-α (TNF-α), and this association typically occurs in a ligand-dependent manner. Nuclear factor (NF)-κB, positioned downstream of the TLR4 signaling pathway, plays a pivotal role in orchestrating immune responses, cellular proliferation, and differentiation. Upon activation, TLR4 induces nuclear translocation of NF-κB (p65), thereby facilitating the expression of diverse inflammatory cytokines, such as IL-1β and TNF-α^19^. The activation of the TLR4/NF-κB signaling pathway facilitates the assembly of a complex between NLRP3 and apoptosis-associated speck-like protein with a CARD (ASC), which subsequently interacts with the cysteine protease caspase-1 to form inflammasomes^20,21^. The classic pyroptotic pathway, mediated by the NLRP3 inflammasome, is pivotal in determining functional outcomes following stroke^22^.

Recently, a novel RCD pattern, distinct from the well-known RCD pattern, has been identified and designated as “cuproptosis”. This unique mode of cell death relies on the copper (Cu)-mediated targeting of lipoacylated tricarboxylic acid (TCA) cycle proteins and is strongly associated with mitochondrial respiration^23^. FDX1 converts Cu (II) to the highly toxic Cu (I), resulting in the aggregation of fatty acylated proteins, exhaustion of iron-sulfur cluster proteins, HSP70 activation, and induction of intracellular toxic oxidative stress, ultimately causing cell death. Importantly, this process is associated with mitochondrial respiration. A meta-analysis has revealed that serum Cu levels are substantially elevated during the acute phase of stroke^24^. Recent evidence suggests that Cu ions participate in various transformation mechanisms that damage brain tissue during fusion injury^25^. The induced cuproptosis activates HSP70 to initiate an immune inflammatory response.

Disulfiram (DSF) is a well-established anti-alcoholic medication scientifically validated for its safety and potential in targeted tumor therapy^26,27^. Recent research has demonstrated the potential of DSF in inhibiting multiple inflammatory reactions and regulating inflammation-related targets, highlighting its potential as an anti-inflammatory agent^28–30^. The DSF-Cu complexes were found to be safe and efficacious in the prevention and treatment of various types of cancers^31^. The inclusion of DSF in the Cu carrier category aimed to enhance the induction of apoptosis in tumor cells^23^. Recently, Cu (II) bis (diethyldithiocarbamate), a potent anticancer agent, has been identified as a bioactive metabolite of alcohol abuse drug disulfiram^32^. The administration of this drug was shown to reduce the expression of FDX1 an upstream regulator involved in mitochondrial proteolipid acylation during cuproptosis^33^. Further comprehensive investigations are warranted to ascertain the potential of DSF in ameliorating CI/RI damage and to elucidate the underlying mechanisms. Therefore, in the present study, we aimed to validate the effect of DSF on CI/RI and elucidate the underlying molecular mechanisms. We hypothesized that DSF could modulate Fdx1-induced CI-RI damage and inhibit inflammatory responses. We believe that the findings of the current study would further unravel the crosstalk relationship between each death mechanism and provide new targets for drug application and treatment of CI-RI.

## Experimental methods

### Animals and tMCAO models

Male C57BL/6 mice (6–8 weeks old, weight 20 ± 3 g) were procured from Liaoning Changsheng Biotechnology Co., Ltd and housed in a hygienic and comfortable habitat under a 12 h natural light–dark cycle to simulate their circadian rhythm. The Animal Experiment Center of Harbin Medical University provided adequate food and water. The animals were randomly divided into four groups (n = 4/group): sham group, 24 h-tMCAO group, DSF + 24 h-tMCAO group, and vehicle + 24 h-tMCAO (dimethyl sulfoxide < 2%). The DSF + 24 h-tMCAO group was intraperitoneally administered DSF (50 mg/kg, twice daily), starting from the day before the surgery and continuing postoperatively. The mice were anesthetized with 2% isoflurane, and body temperature was maintained at ~ 37 ℃ throughout the surgical and postoperative periods using a servo-controlled heating blanket. Focal cerebral ischemia was achieved by occluding the middle cerebral artery (MCA). After performing a midline incision in the neck, a doccol suture (602256PK5Re; US) was inserted into the right external carotid artery and then reversed into the right internal carotid artery via the common carotid artery to occlude the blood supply to the MCA. The sutures were then removed after 1 h.Following suture removal, the mice exhibited recovery times of 6 h, 24 h, 3 d, 5 d and 7 d. Mice died of cervical dislocation after anesthesia at the above time points. This experiment was reviewed and approved by the ethical review committee of Harbin Medical University. (IACUU NO.2022133).The management and use of mice are consistent with the relevant guidelines of US National Institutes of Health.

### Neurological function assessment

Longa’s method^34^ was used for blind evaluation before sampling, which was divided into 5 grades: grade 0, no neurological dysfunction; Grade 1, inability to extend the forepaw flexion; Grade 2, hover to the paralyzed side; Grade 3, fall or even tumble; Grade 4, inability to walk and poor consciousness.

### 2,3,5-Triphenyltetrazolium chloride (TTC) staining

Briefly, the harvest mouse brain was sliced into seven pieces (1 mm thick) from the rostral end of the frontal lobe. Cells were then incubated in TTC (Solarbio, Beijing, China), shielded from light for 30 min, and fixed in 4% paraformaldehyde. Planar infarct volume measurements were performed using ImageJ software (National Institutes of Health, Bethesda, MD, USA).

### Hematoxylin–eosin and Nissl stainings

The specimens were fixed in a 4% paraformaldehyde solution and subsequently embedded in paraffin. Histological sections (4 µm thick) were prepared and stained with hematoxylin–eosin. For Nissl staining, a 1% toluidine blue solution was heated to 60 ℃, followed by the immersion of all sections for 40 min. Subsequently, sections were washed with 70% ethanol and rapidly differentiated using 95% ethanol. The tissue sections were examined and imaged under a light microscope (Nikon, Tokyo, Japan).

### TUNEL assay

One-step TUNEL In Situ Apoptosis Kit (Elabscience, E-CK-A321) was used to detect brain cell apoptosis in each group. Specific DNA endonucleases were activated during apoptosis. The TdT enzyme ligated the luminous dUTP to the broken DNA and was found under a silver light microscope.

### Reactive oxygen species production

Using fluorescent probe DCFH—DA detection of reactive oxygen species, into the intracellular DCFH—DA were hydrolyzed to DCFH DCF model combined with reactive oxygen species generated glow.

### Immunofluorescent staining

Briefly, the prepared brain tissues were sequentially immersed in a 4% paraformaldehyde solution and 30% sucrose solution for 48 h. After embedding in optimal cutting temperature compound and freezing, frozen blocks were sectioned (7 µm thick slices) using a freezing microtome. Sections were treated with a blocking solution (Beyotime, China) containing Triton X-100 for 20 min and incubated overnight at 4 ℃ with the following primary antibodies: ASC (sc-514414, 1:50 dilution , Santa Cruz, USA), Caspase-1 (sc-56036, 1:50 dilution, Santa Cruz, USA), GSDMD (sc-393581, 1:50 dilution, Santa Cruz, USA), HSP70 (T55496, 1:50 dilution, Abmart, China). Following hydrations with PBST (3 ×), the samples were incubated with the appropriate fluorophore-conjugated secondary antibody (Boster, China) for 1 h. Nuclei were stained with 30 μL DAPI (Abcam, UK) to ensure no bubbles. After 10 min, the slides were examined using light microscopy.

### Immunohistochemical staining

The fixed brain tissues were embedded in paraffin, sectioned, deparaffinized, and subjected to antigen retrieval using a citrate solution after blocking with 3% hydrogen peroxide. The sections were then blocked with fetal bovine serum for 1 h. Subsequently, the sections were incubated overnight at 4 ℃ with primary antibodies against FDX1 (T510671,1:200 dilution, Abmart, China), DLST (TD13671,1:200 dilution, Abmart, China), ATP7A (PA7106, 1:200 dilution, Abmart, China), ATP7B (TA0410, 1:200 dilution, Abmart, China), Caspase-1 (22915-1-AP, 1:200 dilution, Proteintech, USA), and IL-18 (10663-1-AP, 1:200 dilution, Proteintech, USA), followed by subsequent incubation with secondary antibodies (Boster, China) for 1 h at room temperature. Color development was achieved using diaminobenzidine. Then, sections were stained with hematoxylin. Subsequently, the sections were dehydrated and sealed before imaging under a light microscope at 200 × magnification. The acquired images were analyzed using ImageJ software to determine the average density within the observation area.

### Cu and iron levels detection

Cu and iron levels were detected using colorimetric quantitative kits provided by Nanjing Jiancheng BioEngineering Institute (E010-1-1, A039-2-1, Jiangsu, China). The right brain tissue was measured, followed by the addition of double-distilled water at a ratio of 1:9 and supernatant collection by ultrasonic centrifugation. For protein quantification, the absorbance was measured using a microplate reader (MD, Shanghai, China) after the sequential addition of reagents.

### Western blot analysis

Brain tissue from the right hemisphere was lysed (RIPA: protease inhibitor: phosphatase inhibitor, 100:1:1; lipoacylated protein was added in an equal proportion of TCEP). To determine the sample protein concentration, the supernatant was denatured by boiling, using the BCA protein assay kit (Beyotime, China). Proteins were subjected to sodium dodecyl-sulfate polyacrylamide gel electrophoresis and subsequently transferred onto polyvinylidene fluoride membranes using the sandwich method. Then, the membranes were blocked with 5% skim milk for 1 h at room temperature, followed by overnight incubation with primary antibodies at 4 ℃ in a refrigerator. The membranes were incubated with goat anti-mouse and anti-rabbit secondary antibodies (Abmart, China) at room temperature for 60 min. Next, the membranes were washed with Tris-buffered saline with 0.1% Tween^®^ 20 detergent and incubated with an enhanced chemiluminescence reagent (Biosharp, China) for detection. The primary antibodies employed were as follows: GSDMD (EPR20859, 1:1000, Abcam, UK); Caspase-1 (22915-1-AP, 1:1000 dilution, Proteintech, USA), ASC (sc-514414, 1:500, Santa Cruz, USA),IL-1β(RM1009, 1:1000, Abcam, UK), IL-18 (10663-1-AP, 1:1000 dilution, Proteintech, USA), IL-17 (26163-1-AP, 1:1000, Proteintech, USA),TLR4 (66350-1-Ig, 1:1000dilution, Proteintech, USA), NLRP3(EPR23073-96, 1:1000, Abcam, UK),TNFα (60291-1-Ig, 1:1000 dilution, Proteintech, USA), LIAS (67298-1-Ig, 1:1000 dilution, Proteintech, USA), NF-κB (66535-1-Ig, 1:1000 dilution, Proteintech, USA) and SLC31A1 (CTR1,T510261, 1:1000 dilution, Abmart, China), ATP7B (TA0410, 1:1000 dilution, Abmart, China), HSP70 (T55496, 1:1000 dilution, Abmart, China), FDX1(T510671, 1:1000 dilution, Abmart, China), SDHB (EPR10880, 1:50000 dilution, Abcam, UK), DLAT (T58125, 1:1000 dilution, Abmart, China), DLST (TD13671, 1:1000 dilution, Abmart, China), BAX (WL01637, 1:800 dilution, Wanleibio, China) and Bcl2 (sc-56018, 1:500 dilution, Santa Cruz, USA). For protein bands, gray values were quantified using ImageJ software.

### Transmission electron microscopy

Brain tissues were cut into cubes (≤ 1 mm^3^) in 2.5% glutaraldehyde solution. After fixation with osmium tetroxide, tissue samples were subjected to repeated dehydration using acetone, followed by the addition of resin overnight at room temperature. The resulting tissue Sections (70 nm) were stained with uranyl acetate and lead citrate and dried for transmission electron microscopy.

### Statistical analyses

All results are presented as the mean ± standard error of the mean. Data were analyzed using analysis of variance, followed by Tukey’s post-hoc test. Statistical analyses were performed using GraphPad Prism version 9.5 (GraphPad Software, Inc., Boston, MA, USA). Statistical significance was set at P < 0.05.

### Ethics statement

The animal experiment was approved by the Animal Management and Use Committee of the First Affiliated Hospital of Harbin Medical University (NO. 2022133) and the management and use of mice are consistent with the relevant guidelines of US National Institutes of Health.the study is reported in accordance with ARRIVE guidelines.

## Results

### 1FDX1 expression is up-regulated in the cerebral infarction area after tMCAO

**Figure 1:**
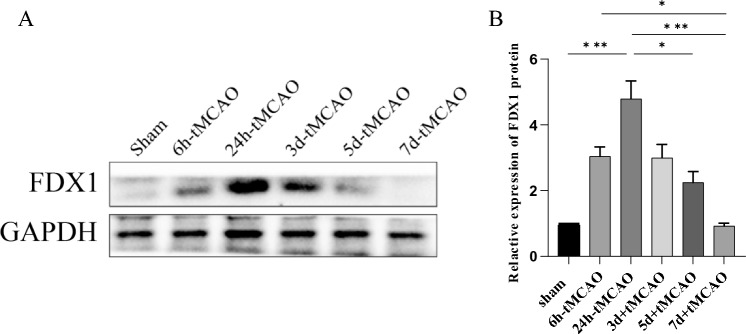
The relative protein levels of FDX1 in brain tissue at different time points after tMCAO were detected by Western blotting. (**A**) The FDX1 protein began to increase significantly on the first day, and then returning to its lowest point. (**B**) Column chart analysis report (***P<0.001, *p<0.05, n = 4) (Original blots are presented in Supplementary Fig. S1).

To explore the relationship between FDX1 and CI/RI, FDX1 protein was determined at different time points after tMCAO. WB showed that FDX1 protein expression reached the peak at 24 h and then gradually decreased compared with Sham group (Fig. 1A,B).

### DSF attenuates the cerebral infarction volume and mitigates neuronal damage following tMCAO

**Figure 2:**
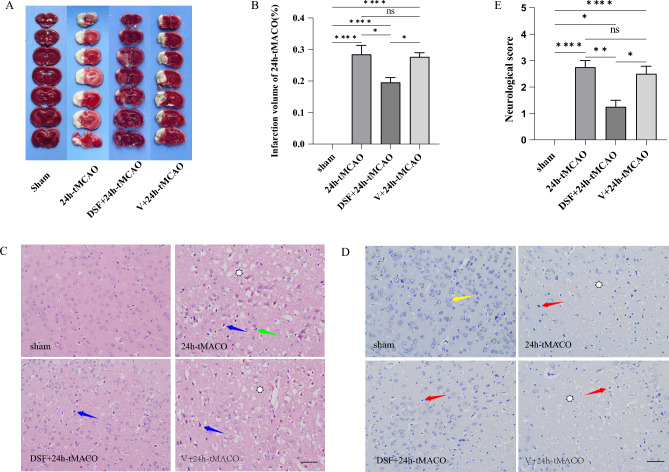
Infarct volume, hematoxylin-eosin staining, and Nissl staining of transient middle cerebral artery occlusion (tMCAO) mice model after disulfiram (DSF) administration (n=4). (**A**,**B**) Representative images and statistical results of TTC staining of brain tissues in different groups. (**C**) Hematoxylin-eosin staining in brain tissue of mice subjected to tMCAO after administration of DSF. The asterisk marks represent the vacuolization of the nerve cells. The green arrow points indicate glial cells and the blue arrow indicates the nucleus of neurons reduced and trachychromatic. (**D**) Nissl staining in brain tissue of mice subjected to tMCAO after administration of DSF. The asterisk marks represent nerve cell vacuolization. The yellow arrow indicates the neuron containing the Niselbosome, and the red arrow indicates the disappearance of the neuron Niselbosome. (**E**) Neurological deficit scores were assessed in mice at 24 h post-tMCAO. *P <0.05; **P < 0.01; ****P <0.0001; and *ns* not significant. Scale bar = 100 μm.

TTC staining confirmed the DSF-mediated neuroprotective effect, reducing CI/RI. Compared with the 24 h-tMCAO group, the DSF + 24 h-tMCAO group exhibited a significant decrease in the infarct volume. (Fig. 2A,B) The neurological deficit score of DSF treatment group was lower than that of 24 h-tMCAO group (Fig. 2E) The microstructural characteristics of the brain tissue were observed following tMCAO. Hematoxylin and eosin staining revealed that tMCAO damaged the area of cerebral infarction and the surrounding tissues. The nerve cell stroma appeared loose and edematous, whereas neuronal nuclei displayed pyknosis and hyperchromatism. Additionally, vacuolation was observed in the neurodermal membrane, along with gliosis. In the DSF + 24 h-tMCAO group, pyknosis and hyperchromatism of neurons were observed in the infarct area, while neuropil vacuolation in the infarct focus was alleviated, with a reduction in the number of unidentified structures and glial cells surrounding the infarct (Fig. 2C). Based on Nissl staining, Nissl bodies, enhanced neuronal activity. Disappeared in neurons after CI/RI, and a large number of damaged neurons with karyopyknosis and dark staining were observed in and around the infarct area. DSF administration preserved normal neuronal morphology in most mice, with most cells displaying visible Nissl bodies. Only a small number of injured neurons with pyknosis and dark staining were observed around the infarction (Fig. 2D).

### DSF mitigates neuronal apoptosis at 24 h post-tMCAO

**Figure 3:**
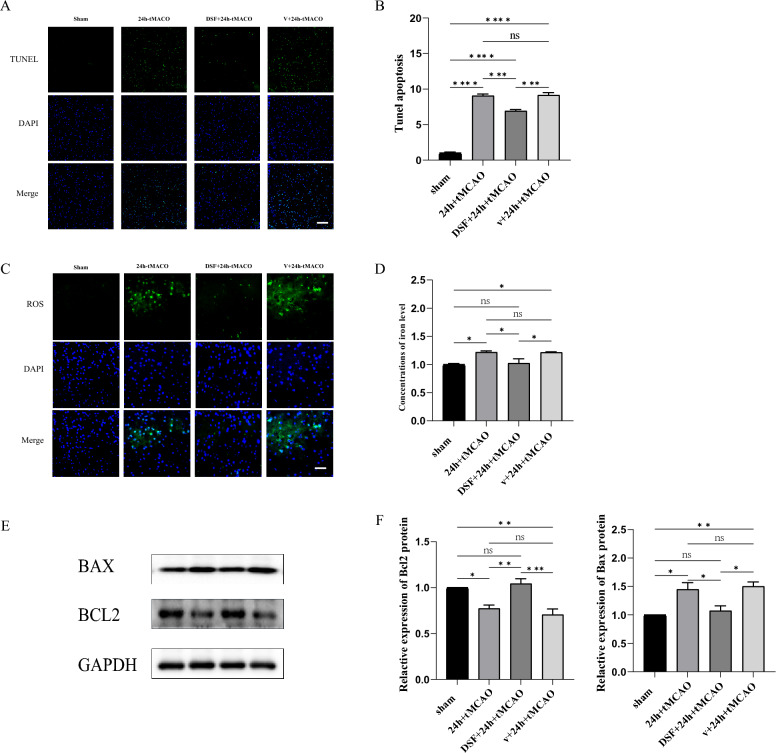
TUNEL assay was used to detect neuronal apoptosis. (**A**) DSF inhibited apoptosis in tMCAO mice (marked in green). Scale bar = 50 μm (n=4). (**B**) Neuronal apoptosis rates in each group. (**C**) Reactive oxygen species (ROS) was detected by fluorescent probe DCFH-DA. Scale bar=100 μm. (**D**) The iron content was determined by colorimetry (n=4). (**E**) Western blot analysis of apoptosis-related proteins and bar graph (**F**) (n=4). *P < 0.05; **P < 0.01; ***P < 0.001; ****P < 0.0001; and *ns* not significant (Original blots are presented in Supplementary Fig. S2).

TUNEL assay showed that the apoptosis of neurons caused by reperfusion injury was obvious, which was significantly improved after DSF treatment (Fig. 3A,B). The expression of BAX and Bcl2 was further verified by WB (Fig. 3E,F). Iron concentrations were reduced in the treatment group (Fig. 3D).

### DSF modulates key factors associated with cuproptosis

**Figure 4:**
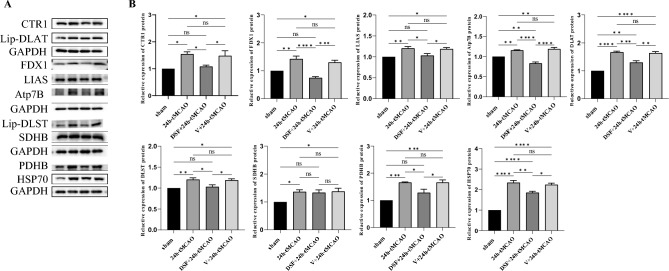
DSF regulates the protein related to cuproptosis (**A**) Contents of cuproptosis-related proteins were determined using western blotting. (**B**) Statistical results of each group. (n=4), *P < 0.05; **P < 0.01; ***P < 0.001; ****P < 0.0001; and *ns* not significant (Original blots are presented in Supplementary Figs. S3, S4).

**Figure 5:**
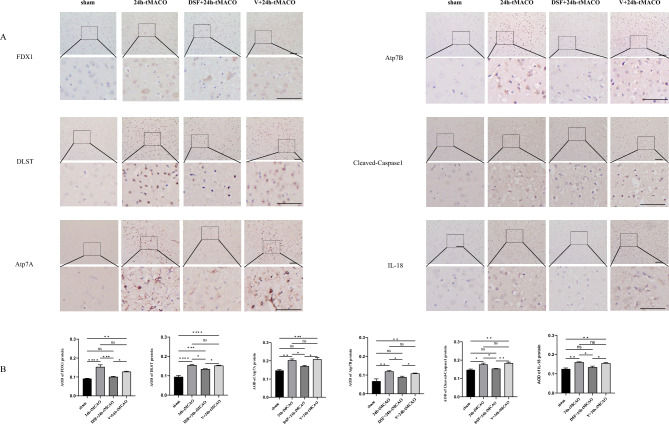
Immunohistochemical staining of the FDX1, DLST, ATP7A, ATP7B, Cleaved-Caspase1and IL-18 protein expression in brain tissues after tMCAO (n = 4). (**A**) The protein expression of each component in the figure exhibited positive results in the 24h-tMCAO model, and subsequently demonstrated a weakened intensity following DSF treatment, which was consistent with the findings from the Western blot experiment. Statistical results of each group (**B**). *P < 0.05; **P < 0.01; ***P < 0.001; ****P < 0.0001; and *ns* not significant. Scale bar = 50 μm.

Functional proteins associated with cuproptosis were determined by western blotting. Induction of tMCAO increased the expression levels of SLC31A1/CTR1, ATP7B, FDX1, and HSP70. Additionally, tMCAO enhanced the levels of crucial proteins associated with the TCA cycle (LIAS, DLAT, DLST, and PDHB). Notably, administration of DSF mitigated the fluctuations observed in these protein levels, SDHB fluctuations are not significant (Fig. 4A,B). Immunohistochemical staining with FDX1, DLST, ATP7A, and ATP7B antibodies revealed that the positive rate of antigen and antibody reaction in DSF + 24 h-tMCAO group was lower than that in 24 h-tMCAO group (Fig. 5A). Based on the results of the semi-quantitative analysis, the expression of the aforementioned proteins was statistically significant (Fig. 5B). Immunofluorescence staining revealed the upregulation of HSP70 expression following tMCAO; however, treatment with DSF mitigated the IR-induced elevation in protein expression DSF regulates the expression cuproptosis-related proteins (Fig. 7A,B).

### DSF inhibits the activation of the HSP70/TLR-4 /NLRP3 signaling pathway and reduces inflammatory cytokines after tMCAO

**Figure 6:**
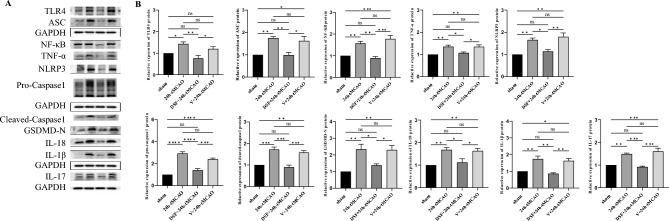
DSF down-regulates proteins involved in inflammatory pathways (n = 4). (**A**) Contents of Inflammatory factors were determined using western blotting. (**B**) Statistical results of each group. *P < 0.05; **P < 0.01; ***P < 0.001; ****P < 0.0001; and *ns* not significant (Original blots are presented in Supplementary Figs. S5–S8).

**Figure 7:**
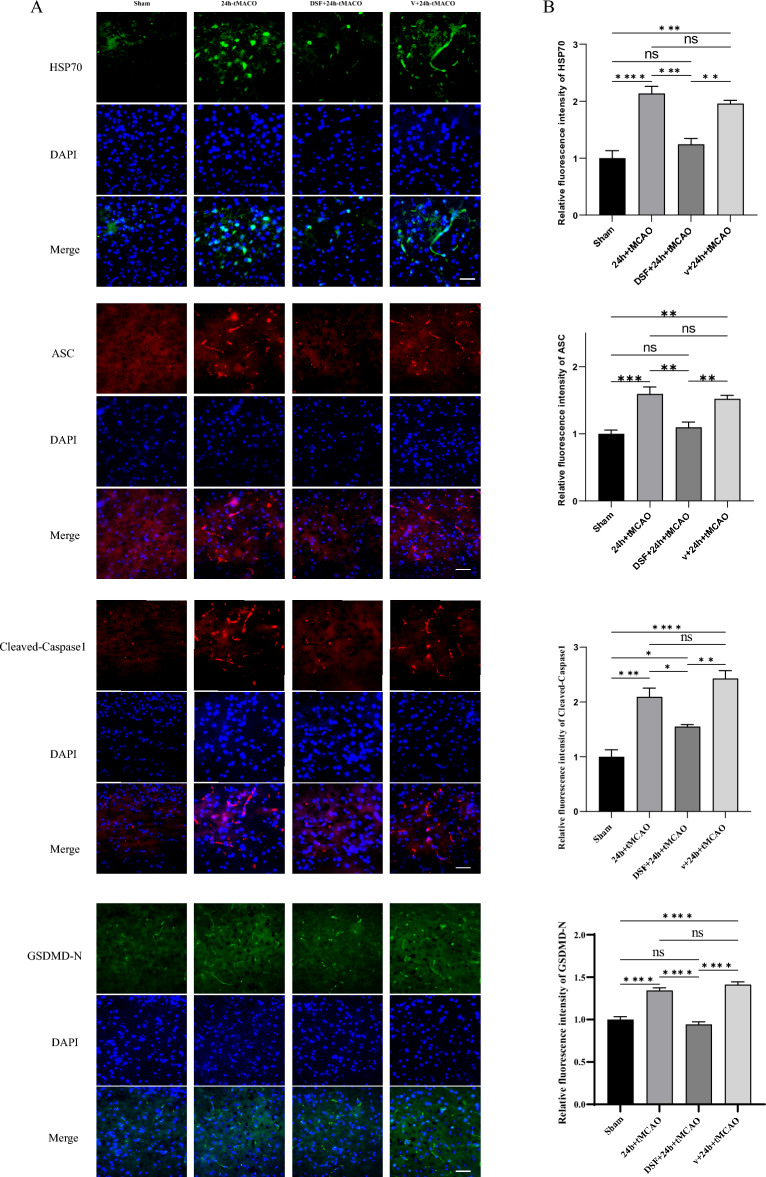
The stress response caused by cuproptosis after cerebral infarction leads to the increase of HSP70, which promotes the occurrence of classical pyroptosis inflammatory response (n = 4). (**A**) Immunofluorescence staining of the expression of HSP70, ASC, Cleaved-Caspase-1 and GSDMD-N in brain tissues. (**B**) Statistical results of each group. *P < 0.05; **P < 0.01; ***P < 0.001; ****P < 0.0001; and *ns* not significant. Scale bar = 50 μm.

DAMPs can activate TLR4 to trigger downstream signaling pathways, and HSP70 is an important endogenous TLR4 agonist. Based on the western blotting analysis, the 24 h-tMCAO group showed increased protein expression levels of TLR-4, NF-κB, NLRP3, TNF-α, ASC, Pro-caspase-1, Cleaved-caspase-1, GSDMD-N, IL-18, IL-1β, and IL-17 when compared with the sham group. However, the DSF-treated group exhibited downregulated protein expression when compared with the control group (Fig. 6A,B). Moreover, immunofluorescence staining revealed that the expression of ASC, caspase-1, and GSDMD-N were upregulated following tMCAO; treatment with DSF could alleviate the reperfusion injury-induced increase in protein expression (Fig. 7A,B). Immunohistochemical staining with IL-18 antibody showed that treatment with DSF decreased positive staining in the 24 h-tMCAO group compared with that in the 24 h-tMCAO group. Based on the results of the semi-quantitative analysis, the expression of the aforementioned proteins was statistically significant in the 24 h-tMCAO group compared with that in the DSF + 24 h-tMCAO group (Fig. 5A,B).

### DSF regulates copper ion homeostasis after tMCAO, protects nerve myelin sheath and relieves mitochondrial oxidative stress in mice

**Figure 8:**
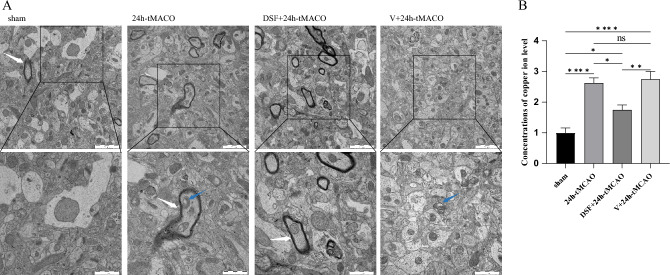
Mitochondrial and myelin morphological changes and copper ion detection. (**A**) Representative transmission electron micrographs showing the brain tissues. Mitochondria (blue arrows) were obviously swollen and cristae was broken and disappeared after tMCAO. Mitochondria were relatively intact in DSF+24h-tMCAO group. Dark black is the nerve myelin sheath (white arrows), 24 h-tMCAO group. The phospholipid layer is loosely wrapped and thinned in thickness. Myelin sheath were relatively intact in DSF+24 h-tMCAO group. (**B**) Copper ion levels decrease with DSF (n = 6). *P < 0.05; **P < 0.01; ***P < 0.001; ****P < 0.0001; and *ns* not significant.

The impact of DSF on Cu levels during CI/RI was assessed by measuring Cu ions. Following CI/RI, the total amount of Cu ions was increased in the 24 h-tMCAO group when compared with that in the sham group, with decrease observed following DSF administration (Fig. 8B). The instability of Cu metabolism leads to mitochondrial morphological damage and dysfunction. Transmission electron microscopy was performed to examine morphological alterations in mitochondria in the brain tissue. The control group exhibited intact mitochondrial membranes and aligned cristae. However, the induction of tMCAO resulted in substantial mitochondrial swelling; most matrixes in the mitochondrial membrane appeared less intense, with a broken crest, which disappeared. The rough endoplasmic reticulum exhibited dilation, degranulation, and vacuolation. The mitochondria in the DSF + 24 h-tMCAO group exhibited intact morphology, suggesting that DSF could suppress mitochondrial fission and preserve mitochondrial structural integrity.The integrity and thickness of the nerve myelin sheath were weakened after ischemia, and the damage of myelin sheath was alleviated after medication (Fig. 8A). DSF treatment improved ROS accumulation in mitochondrial morphological features relative to the 24 h-tMCAO group (Fig. 3C). Compared with the morphology and thickness of myelin sheath in each group, the myelin sheath in the SHAM group was uniform in shape, smooth and complete in appearance, and densely wrapped. In the 24 h-tMCAO group, the myelin sheath in the demyelinated area became discontinuous breaks, dissolved, and the thickness became thinner. The DSF group showed that the integrity and thickness of myelin sheath in the demyelinated area increased, and the wrapping was more compact (Fig. 3A).

## Discussion

Herein, our findings suggest that DSF can exert inhibitory effects on the inflammatory response and cell death induced by CI/RI via the modulation of the TLR4/NLRP3/NF-κB pathway and FDX1/TCA cycle, thereby affording a neuroprotective role. Treatment with DSF alone could suppress the expression of FDX1/TCA cycle-related proteins and the HSP70/TLR4/NLRP3 pathway during CI-RI to protect nerves and inhibit inflammation.

**Figure 9:**
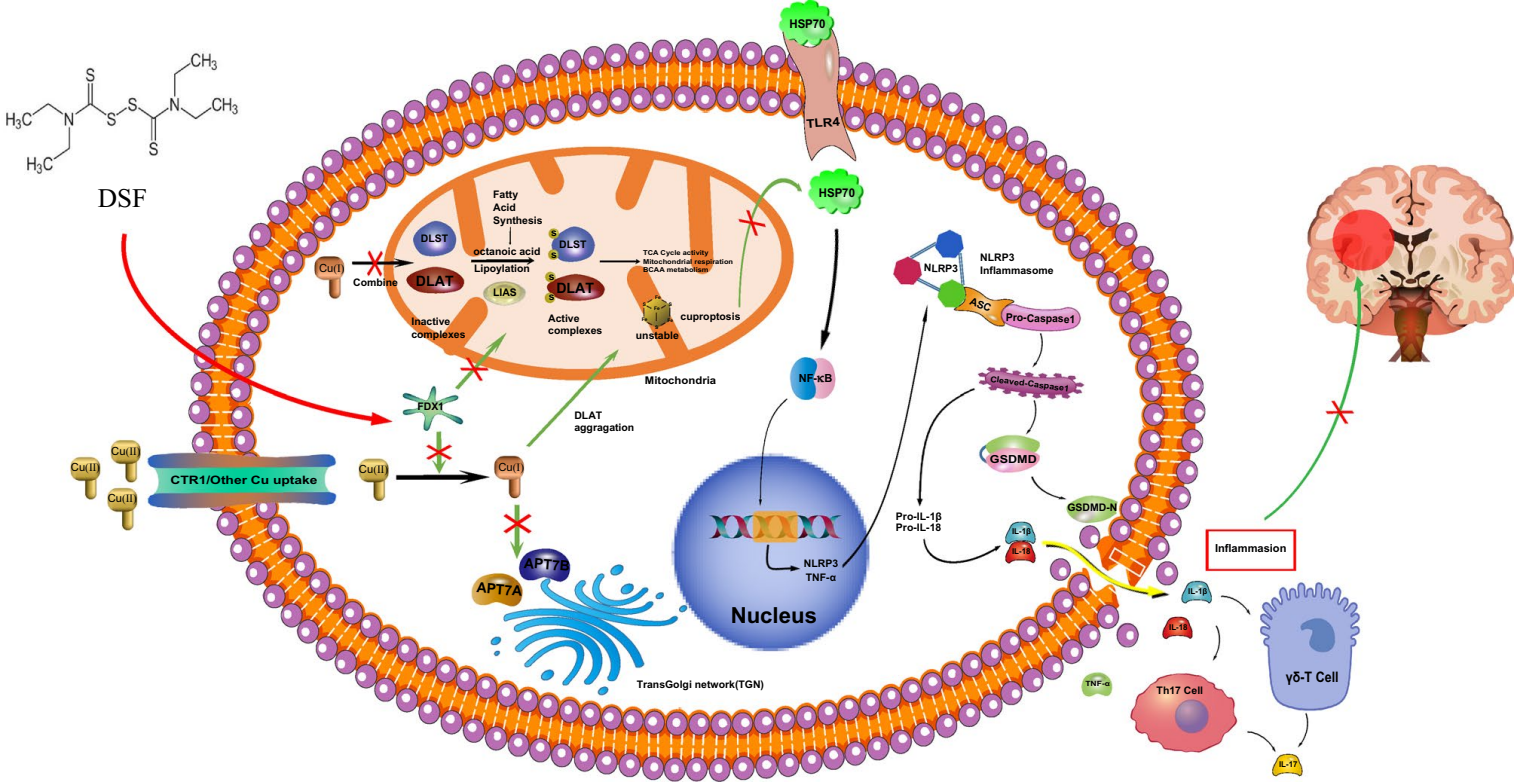
The inflammation of nerve cells in cerebral ischemia-reperfusion injury can be mitigated by Disulfiram through the down-regulation of FDX1.

Cu plays an indispensable role in the physiological processes of the central nervous system. The disruption of Cu homeostasis can exert a multitude of detrimental effects on brain development and function. Compared with other organs, the brain exhibits the highest Cu concentration, second only to the liver. Notably, the average Cu content in the globus pallidus surpasses that in the liver^35^. As a cofactor, Cu plays an indispensable role as a reducing agent in the catalytic activity of peptidyl-glycine alpha-amide monooxygenase, dopamine β-monooxygenase, Cu-zinc (Zn) superoxide dismutase, and ceruloplasmin^36^. The presence of Cu is crucial for catecholamine biosynthesis, neuropeptide activation, and the regulation of mitochondrial function^37^. However, excess Cu induces lipid deposition through oxidative stress, mitochondrial dysfunction^38^ promotes neurodegenerative changes, and cell death^39^ Cu transporters SLC31A1 (CTR1), ATP7A, and ATP7B are known to regulate the Cu content in cellular compartments and maintain Cu homeostasis. CTR1, a master regulator of Cu uptake, acts on the plasma membrane, and the genetic inactivation of CTR1 results in Cu deficiency in cells^40^. In the choroid plexus (ChPl), Cu flows from the epithelial cells of the ChPl into the cerebrospinal fluid (CSF). In patients with Menkes disease, ATP7A inactivation is known to cause Cu deficiency in the brain, numerous metabolic abnormalities (including catecholamine imbalance), delayed neurodevelopment, and death during early childhood^41^. Generally, ATP7B transports Cu from the cytosol into the lumen of the secretory pathway for incorporation into Cu-dependent enzymes and traps excess Cu in vesicles for further export from the cell. The precise impact of these activities on cellular metabolism depends on the specific cell type and can exhibit significant variation across different tissues^42^. Therefore, the negative effects of ATP7B inactivation on brain metabolism are evident in Wilson’s disease. Copper-induced protein transport has been described as a key feature of copper-ATPase, and the central link of this mechanism is the transfer of ATP7A and ATP7B from TGN to peripheral or cytoplasmic vesicles, respectively. The displacement of these proteins is specifically triggered by elevated levels of their own ligand, Cu (I), not Cu (II), and is intended to efflux to restore intracellular copper levels^43–45^ FDX1 helps convert Cu (II) to the more toxic Cu (I), and in our study, DSF inhibited FDX1 expression, reduced Cu (I) levels, and reduced transport of ATP7A and ATP7B. Interestingly, dysregulation of the Cu transport mechanism in the choroid plexus of Atp7b –/– mice has been demonstrated in recent studies; these mice exhibited low Cu levels in the brain at 4 weeks postnatally, emphasizing the crucial role of ATP7B in Cu accumulation^46^. In summary, the inhibition of CTR1 could regulate the cuproptosis pathway by affecting Cu uptake. It is yet to be established whether the upregulation of ATP7A and ATP7B expression following CI-RI serves as a protective mechanism by promoting Cu efflux or accumulation. However, both ATP7A and ATP7B levels and total Cu ion concentration were increased after CI/RI, which is consistent with the anticancer effect of Cu ionophore. This discrepancy can be partly attributed to the limited understanding of the complex mechanisms of action of ATP7A and ATP7B in the brain (Fig. 9).

The immune system relies on Cu to execute certain functions. Recent studies have demonstrated that even in the presence of a marginal deficiency, Cu contributes to a decrease in IL concentrations. Neutrophils accumulate Cu as they differentiate into more mature cell populations, and this accumulation is not reflected by an increase in Cu/Zn superoxide dismutase or cytochrome c oxidase activity^47^ HSP70 is induced by Cu exposure^48^ TLR4 is a PRR that recognizes specific DAMPs such as HSP70, which, in turn, triggers TLR4-mediated inflammatory responses. Thus, HSP70 in the CSF may function as a neuroinflammatory mediator^17^. The inflammasome protein complex has recently emerged as a pivotal component of the innate immune response during ischemic stroke^49^. NLRP3 has been extensively studied in central nervous system diseases^50^. The activation of the NLRP3 inflammasome necessitates TLR4-mediated activation of the p65 subunit within the downstream NFκB pathway^51^. Subsequently, NLRP3 binds to ASC to form a complex, and Caspase-1 specifically cleaves GSDMD, releasing the N-terminus from its self-inhibitory C-terminus. GSDMD-N binds to lipids to form non-selective pores, resulting in cell membrane rupture and promoting the release of a large number of inflammatory factors, ultimately triggering pyroptosis^52^. With the maturation and efflux of IL-18 and IL-1β, Th17 cells and γδT cells release interleukin-17 (IL-17), an important factor in the adaptive immune system^53^. Several studies have shown that IL-17 is associated with the pathogenesis of cerebral ischemia–reperfusion. Loss of γδT cells alleviates brain tissue damage after ischemia–reperfusion, and IL-17 positive lymphocytes are also detected in brain tissue validated in the field of stroke patients^54^. The activated NLRP3 inflammasome promotes the activation of IL-17 in CI/RI^50^.

The anti-inflammatory efficacy of DSF has been substantiated in numerous studies. However, its role in CI-RI remains elusive. In the current study, we found that the association between CI/RI and Cu-induced cell death was accompanied by an enhanced inflammatory response (classic pyroptosis), which was mediated via the HSP70/TLR4/NLRP3 pathway. Importantly, the administration of DSF effectively inhibited this pathway to alleviate CI/RI. Our findings suggest that DSF exhibits robust safety as a well-established pharmaceutical employed in clinical settings for an extensive duration. This finding challenges the conventional belief that DSF, in conjunction with Cu ionophores, forms a complex that effectively treats cancer and induces apoptosis, specifically in cancer cells. However, conventional copper chelators hamper angiogenesis, impeding brain tissue recovery following ischemia. Hence, further investigations into drug applications are warranted. Collectively, the findings of the current study further suggest the presence of potential crosstalk among multiple RCDs in ischemic stroke-induced neuroinflammation, such as pan-apoptosis, potentially mediated via multiple mechanisms.

## Conclusions

The CI/RI model in C57BL/6 mice revealed that the cuproptosis-induced inflammatory pathway contributes to CI/RI and that DSF exerts a protective effect on mitochondria while reducing the cerebral infarct size by inhibiting FDX1-mediated protein esterification and HSP70-mediated inflammatory response. The findings of the current study present a novel concept for enhanced mitigation of cerebral blood flow recanalization-induced damage.

### Supplementary Information

Supplementary Information.

## Data Availability

All data generated or analysed during this study are included in this published article [and its supplementary information files].
