# Supplementary material for: Disulfiram downregulates ferredoxin 1 to maintain copper homeostasis and inhibit inflammation in cerebral ischemia/reperfusion injury
Source: Sci Rep. 2024 Jul 2;14:15175. doi: 10.1038/s41598-024-64981-x (PMC11219760; doi:10.1038/s41598-024-64981-x)

Disulfiram downregulates ferredoxin 1 to maintain copper homeostasis and inhibit inflammation in cerebral ischemia/reperfusion injury.

Shuai Yang[1],Xudong Li[2],Jinhong Yan[2],Fangchao Jiang[1],Xuehui Fan[1], Jing Jin[1],Weihua Zhang[1] ,Guozhong Li[3*]and Di Zhong[1*]

[1] Harbin Medical University, Harbin, China

[2]Fourth Affiliated Hospital of Harbin Medical University, China

[3]Harbin Medical University, Harbin, China;

Heilongjiang Provincial Hospital, Harbin, China

* Correspondence:

lgzhyd1962@163.com（G.Li）

dityan@163.com (D.Zhong)

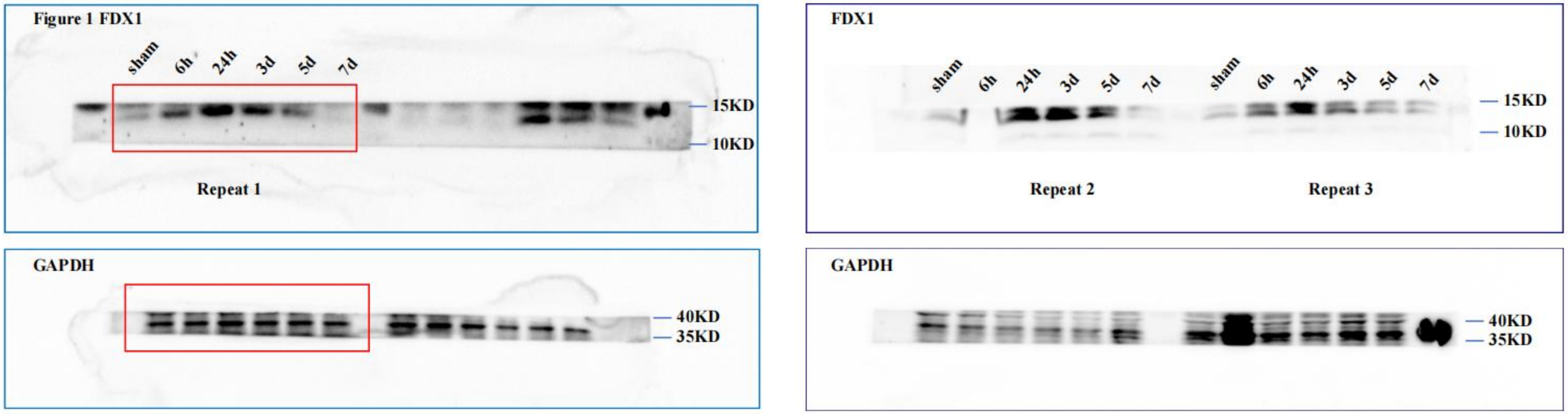

Supplementary Figure S1 The original Western blot in the figure is indicated by the red box.We completed the Western-blot experiment shown in this picture in the same gel. However, in order to incubate different primary antibodies, western blots were cropped prior to incubation with primary antibody hybridization. We repeated the experiment three times on a single gelatin plate.

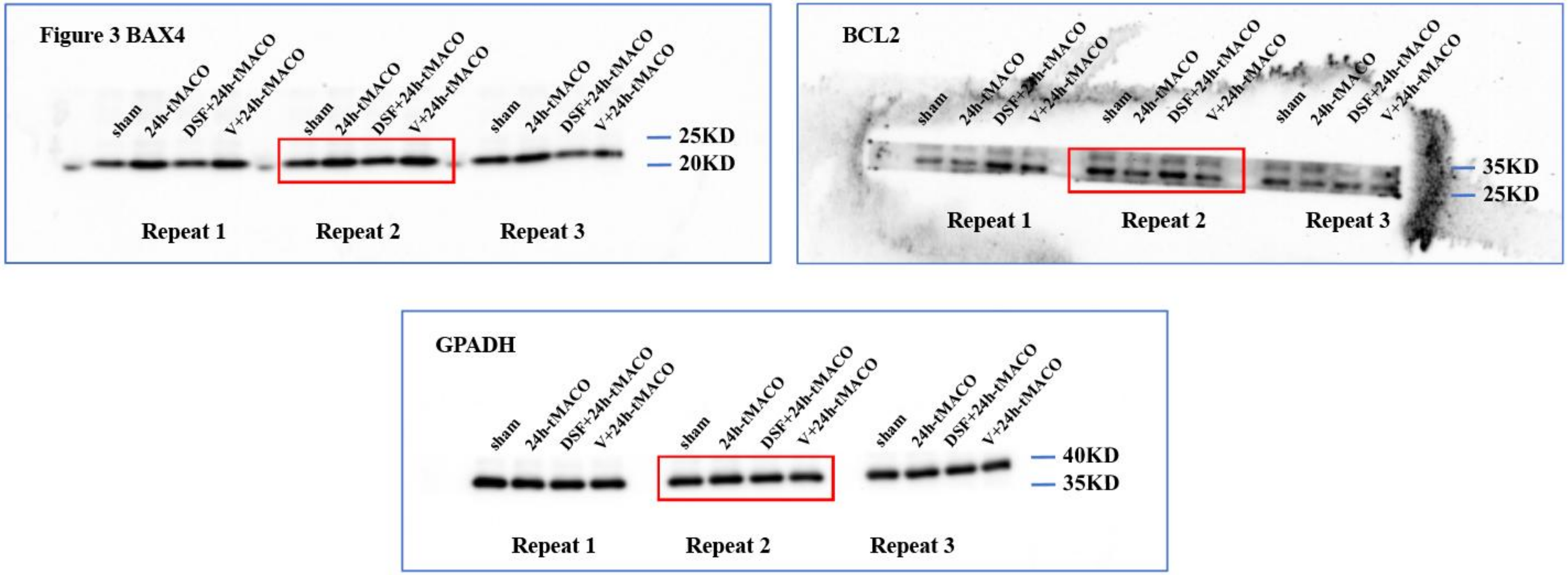

Supplementary Figure S2 The original Western blot in the figure is indicated by the red box.We completed the Western-blot experiment shown in this picture in the same gel. However, in order to incubate different primary antibodies, western blots were cropped prior to incubation with primary antibody hybridization. We repeated the experiment three times on a single gelatin plate.

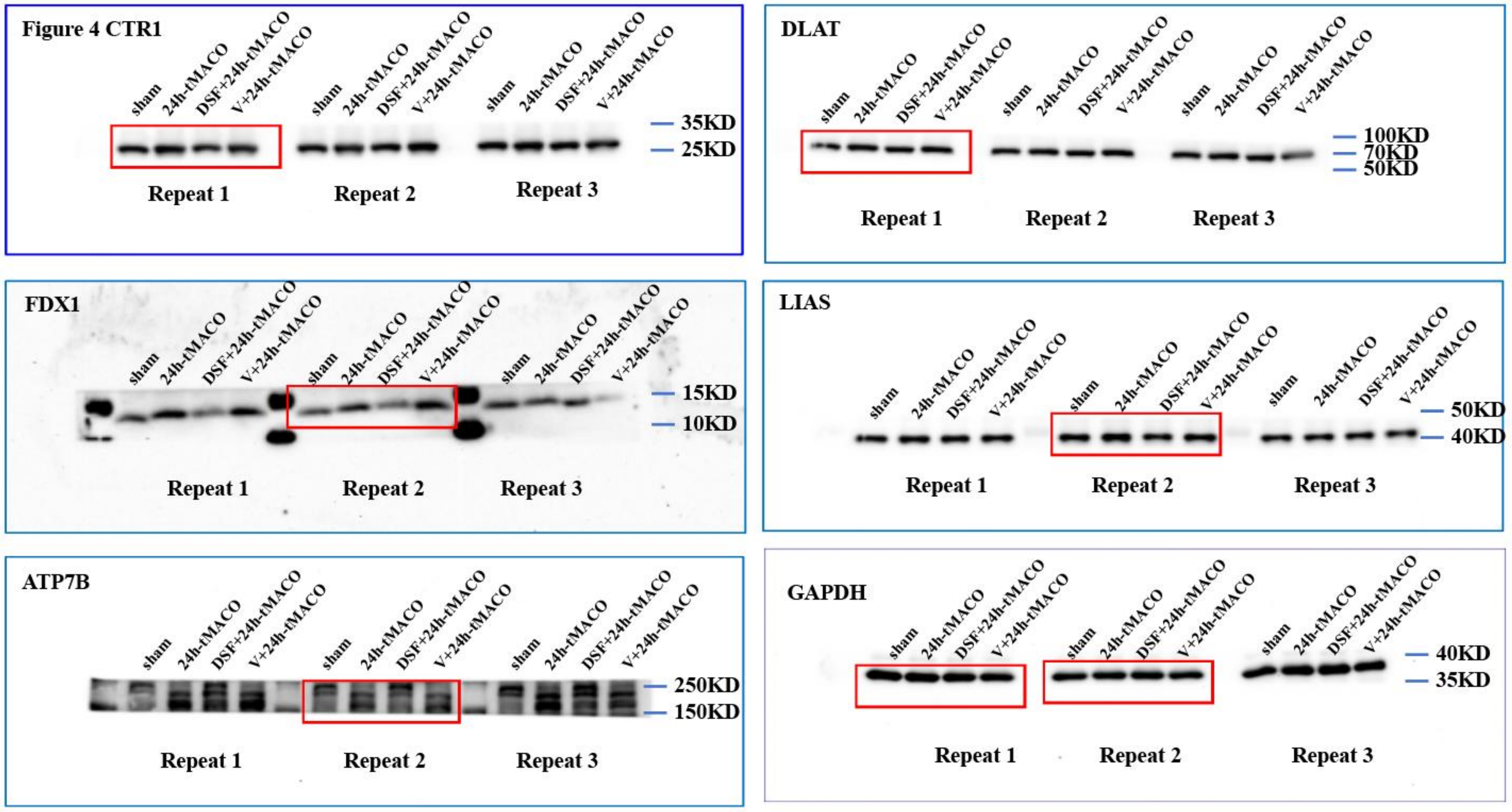

Supplementary Figure S3

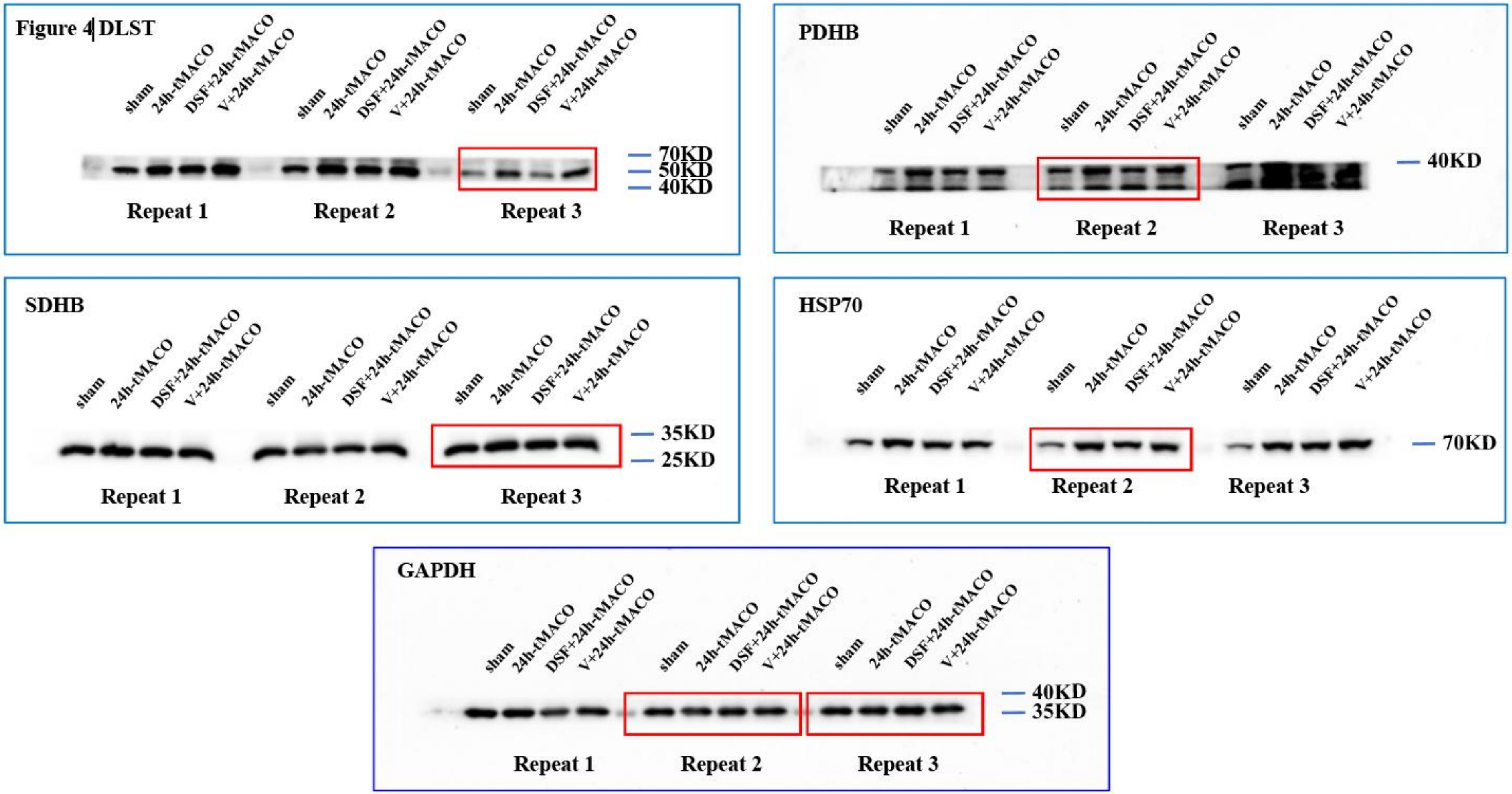

Supplementary Figure S4

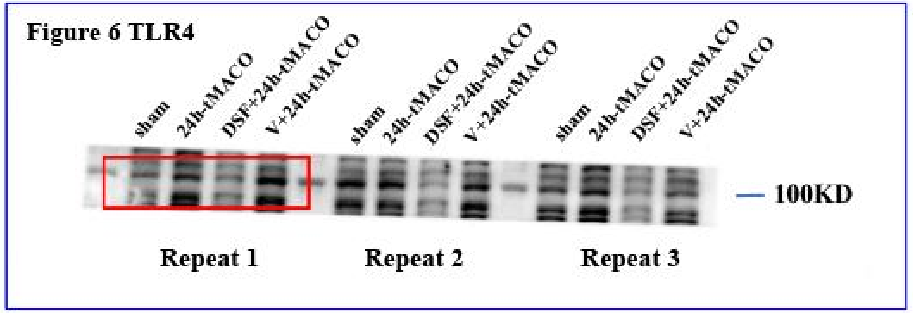

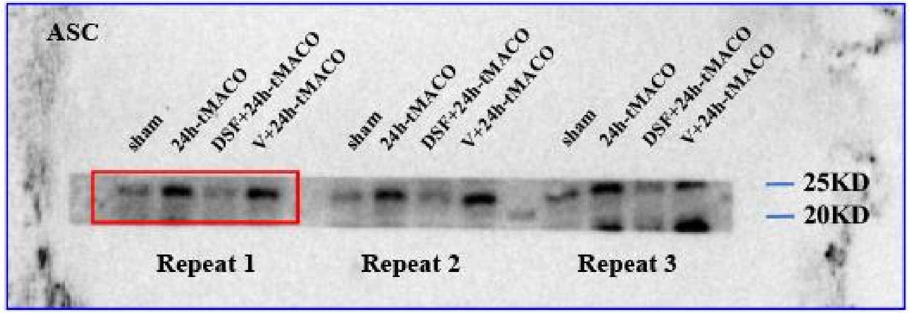

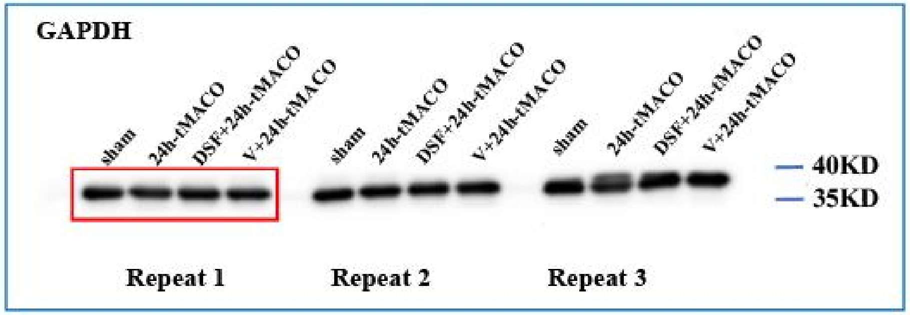

Supplementary Figure S5

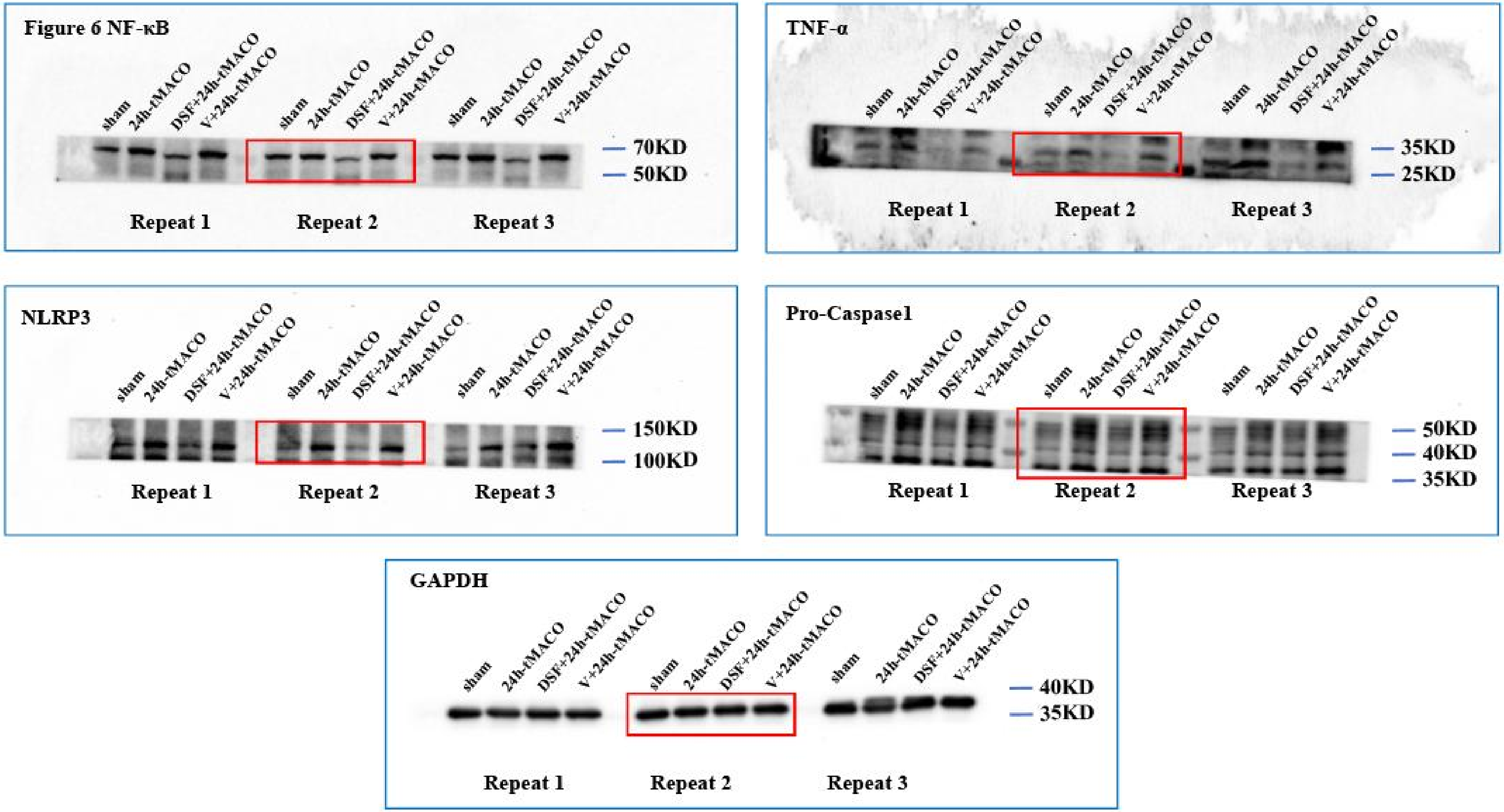

Supplementary Figure S6

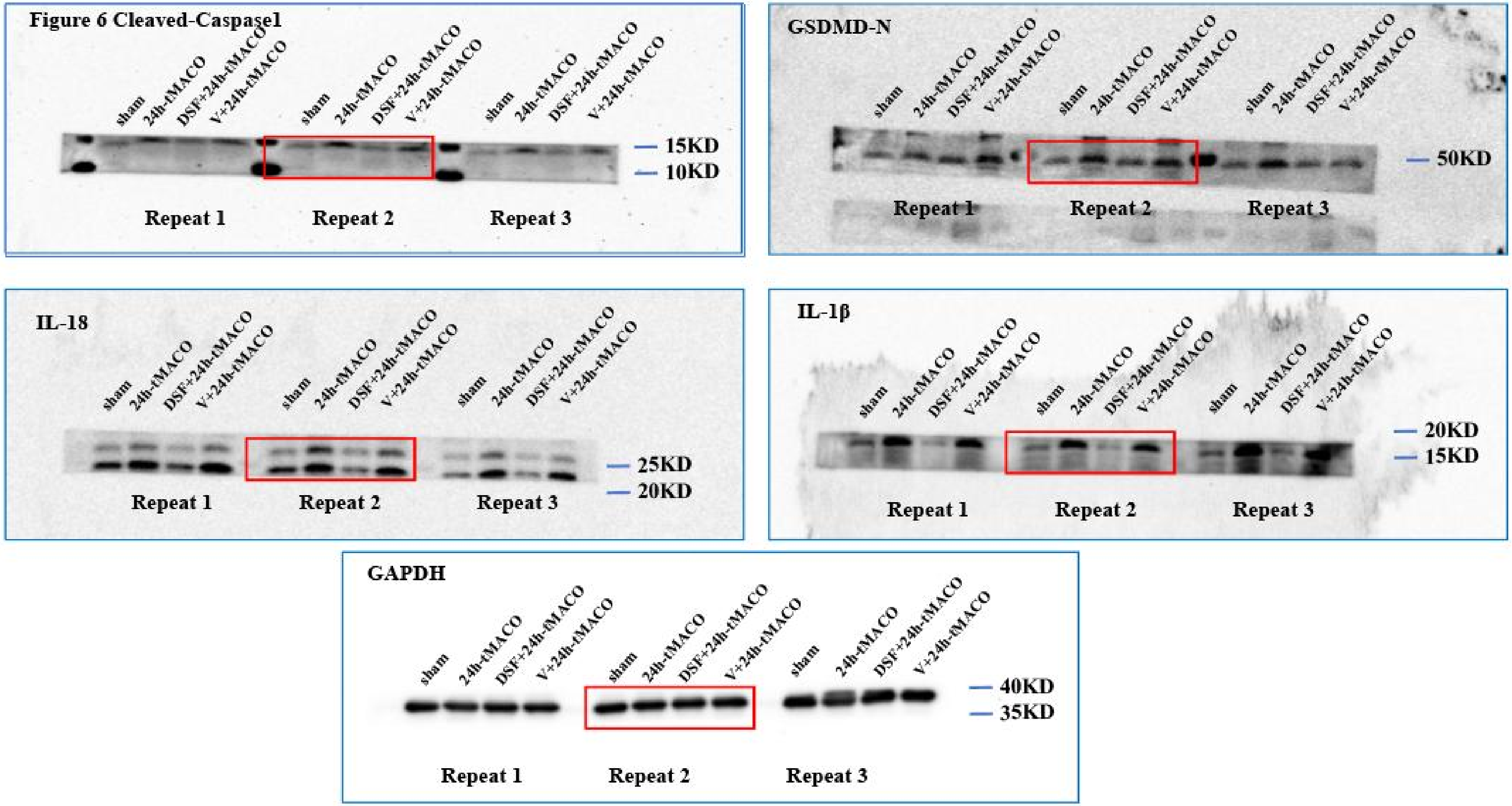

Supplementary Figure S7

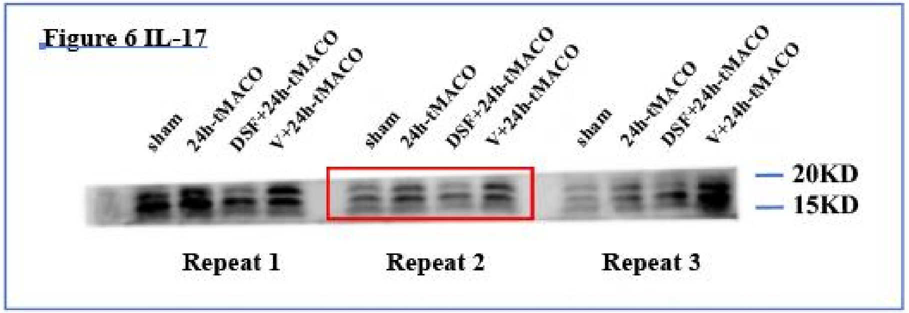

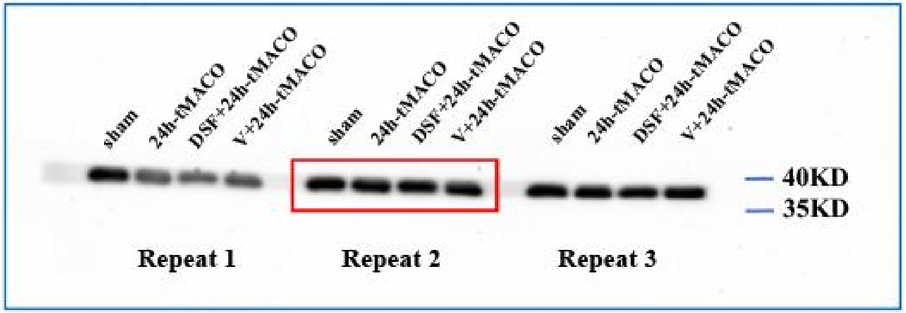

Supplementary Figure S8

Figure 1 FDX1

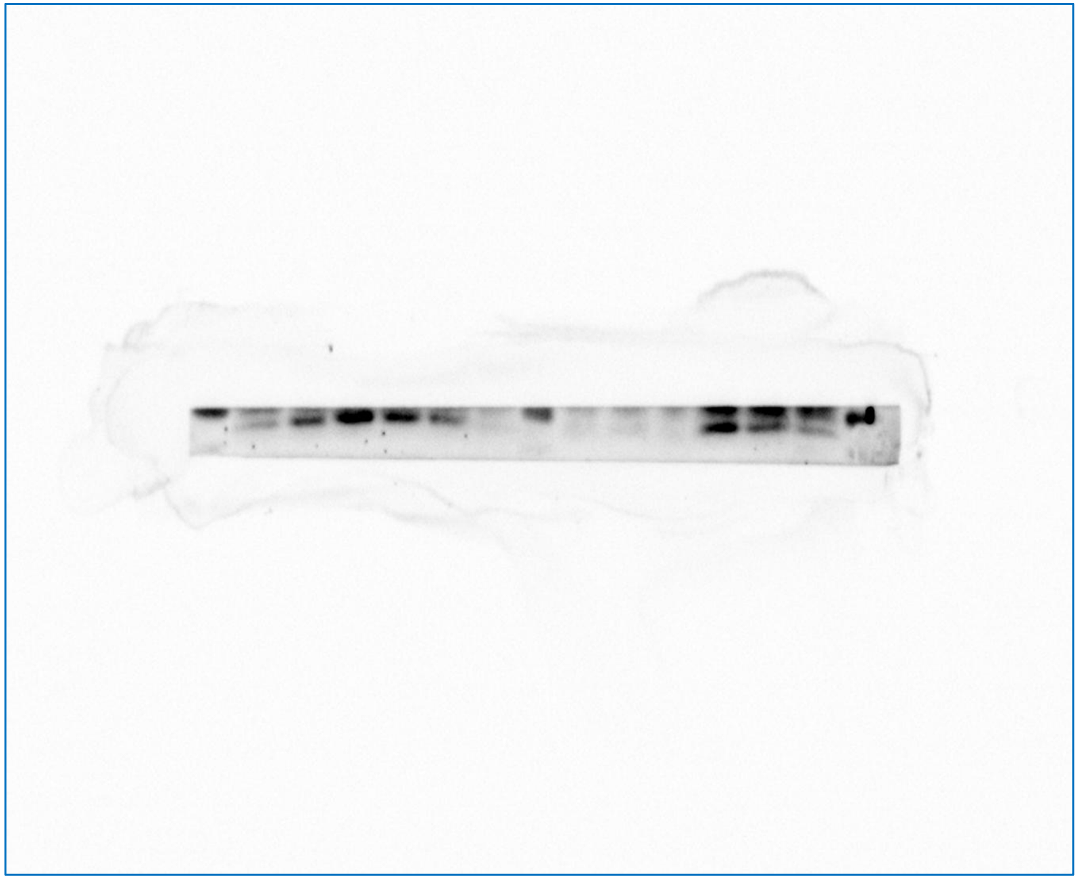

Figure 1 GAPDH

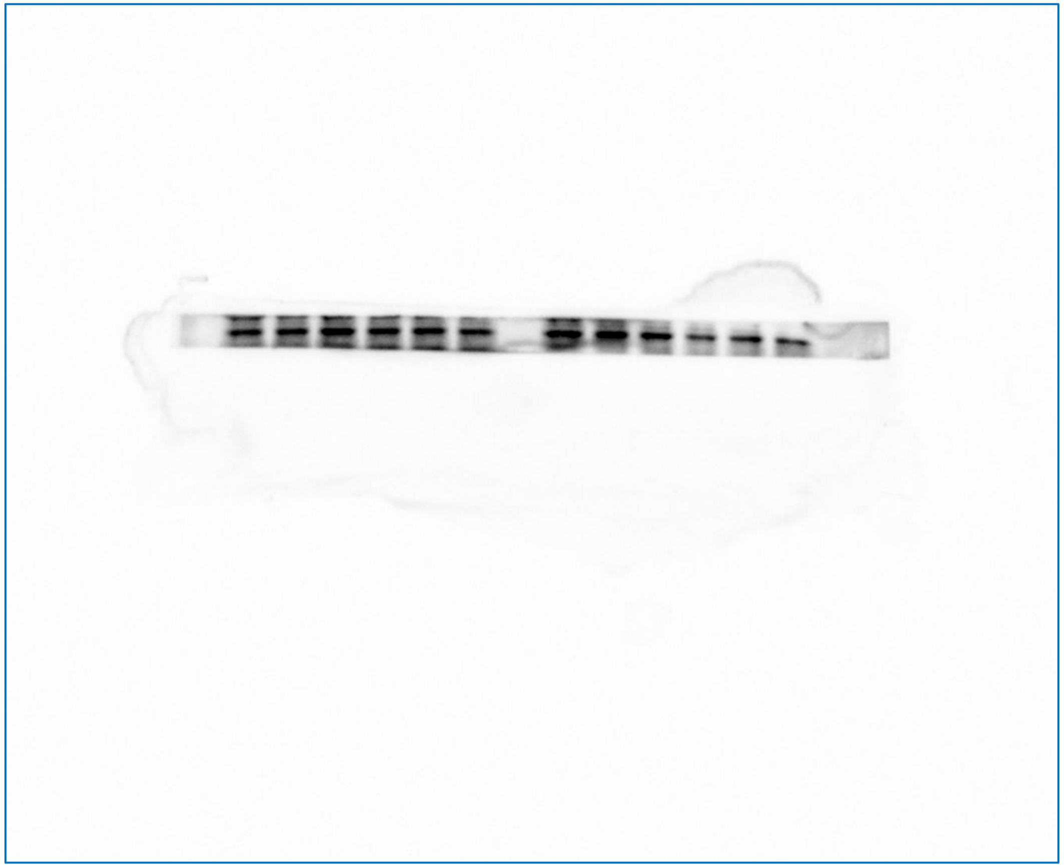

Figure 3 BAX4

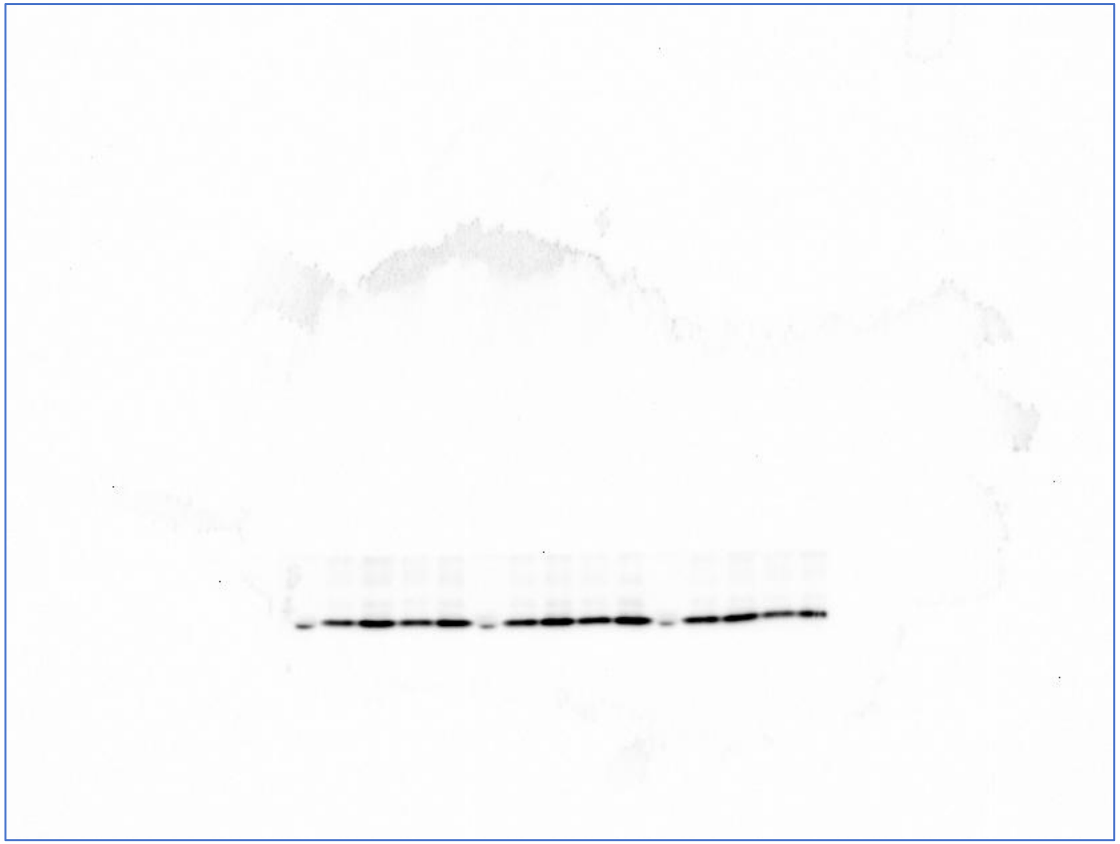

Figure 3 BCL2

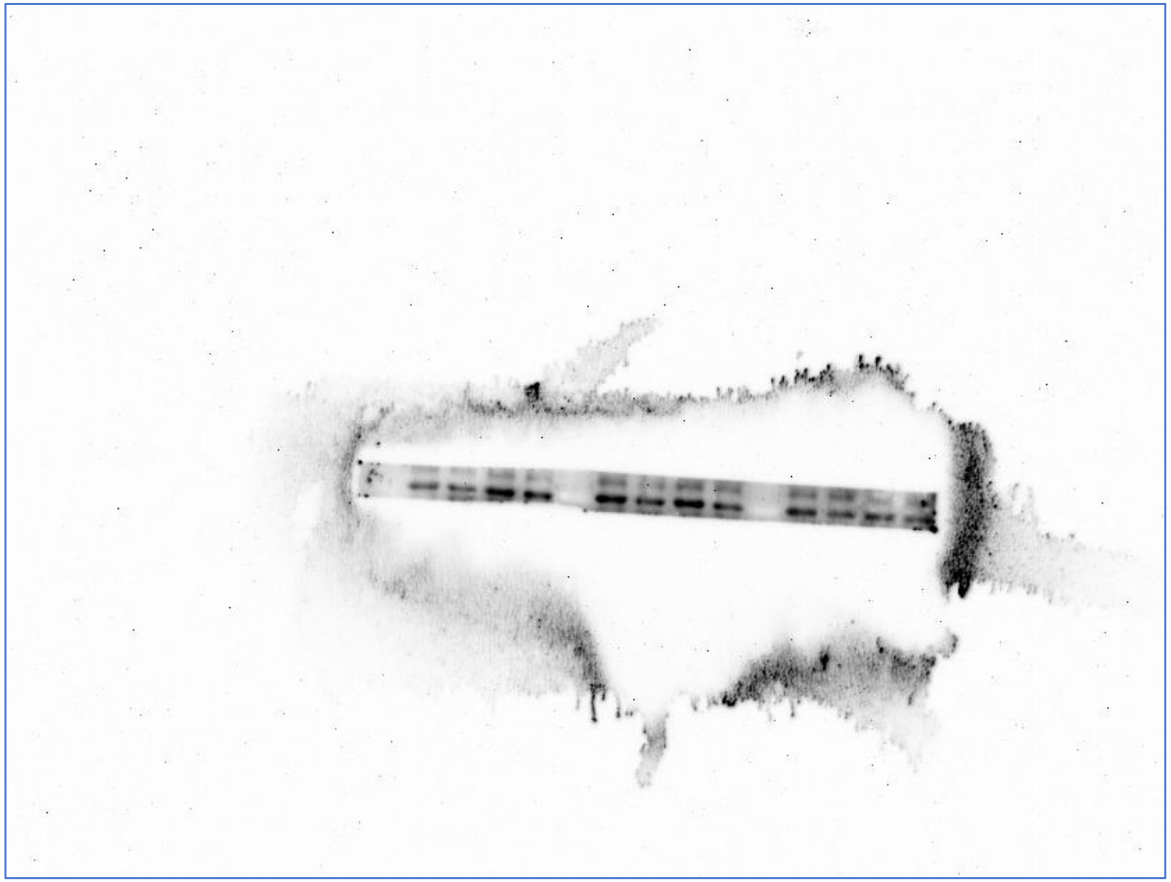

Figure 3 GPADH

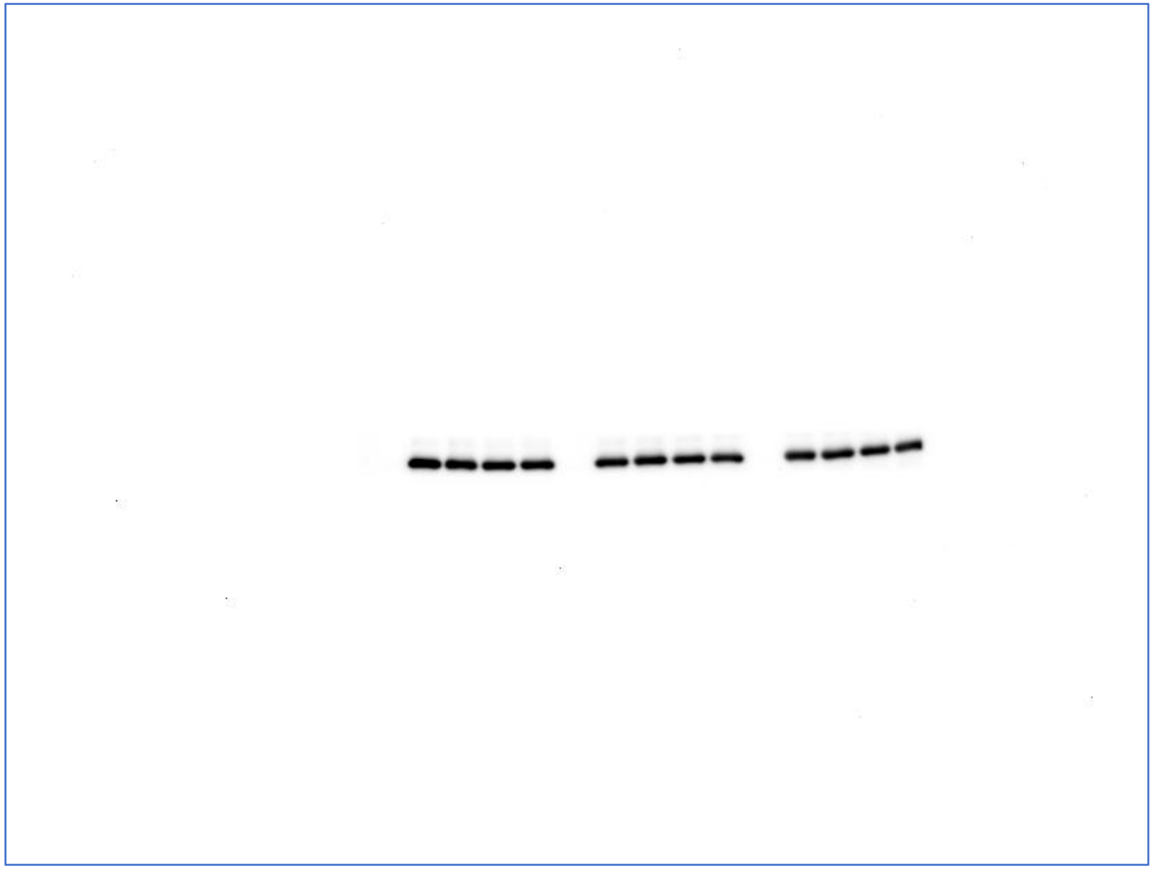

Figure 4 CTR1

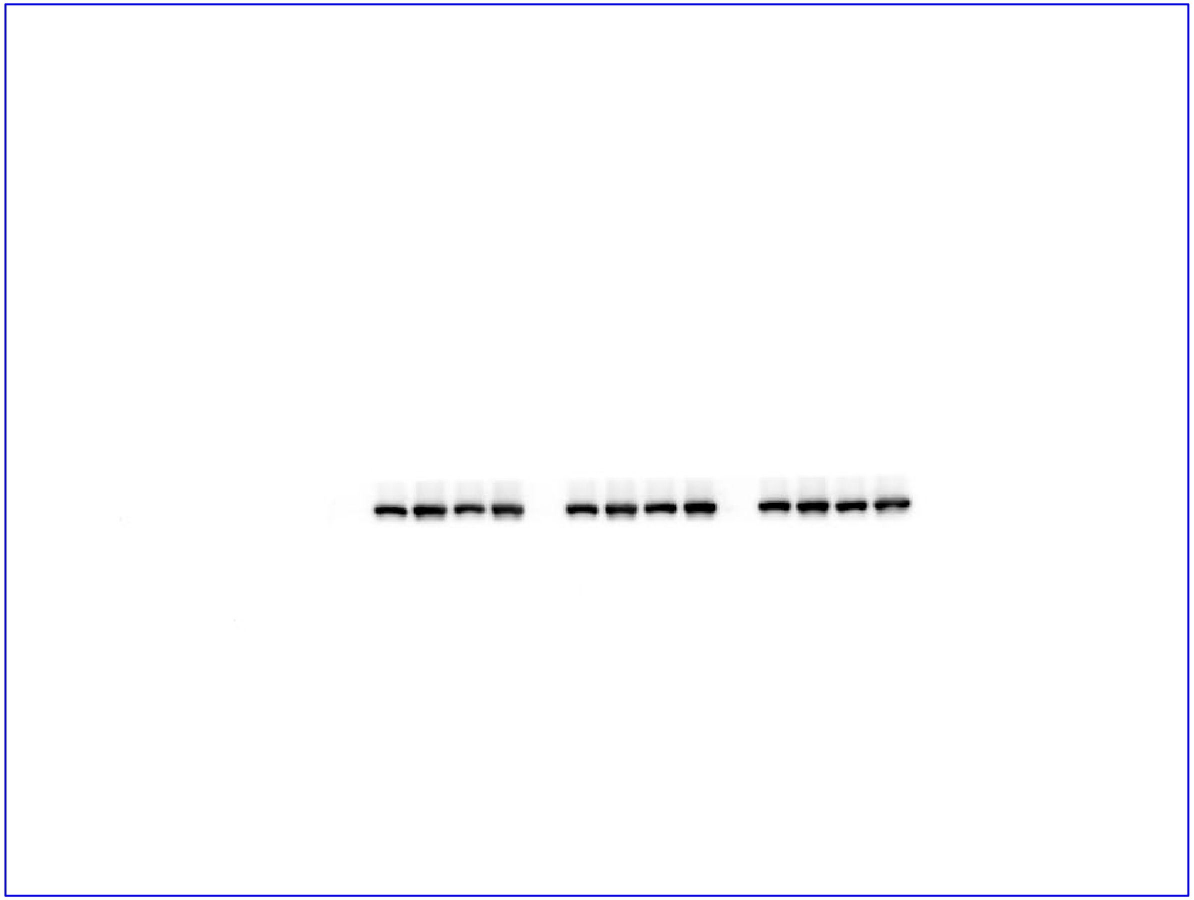

Figure 4 FDX1

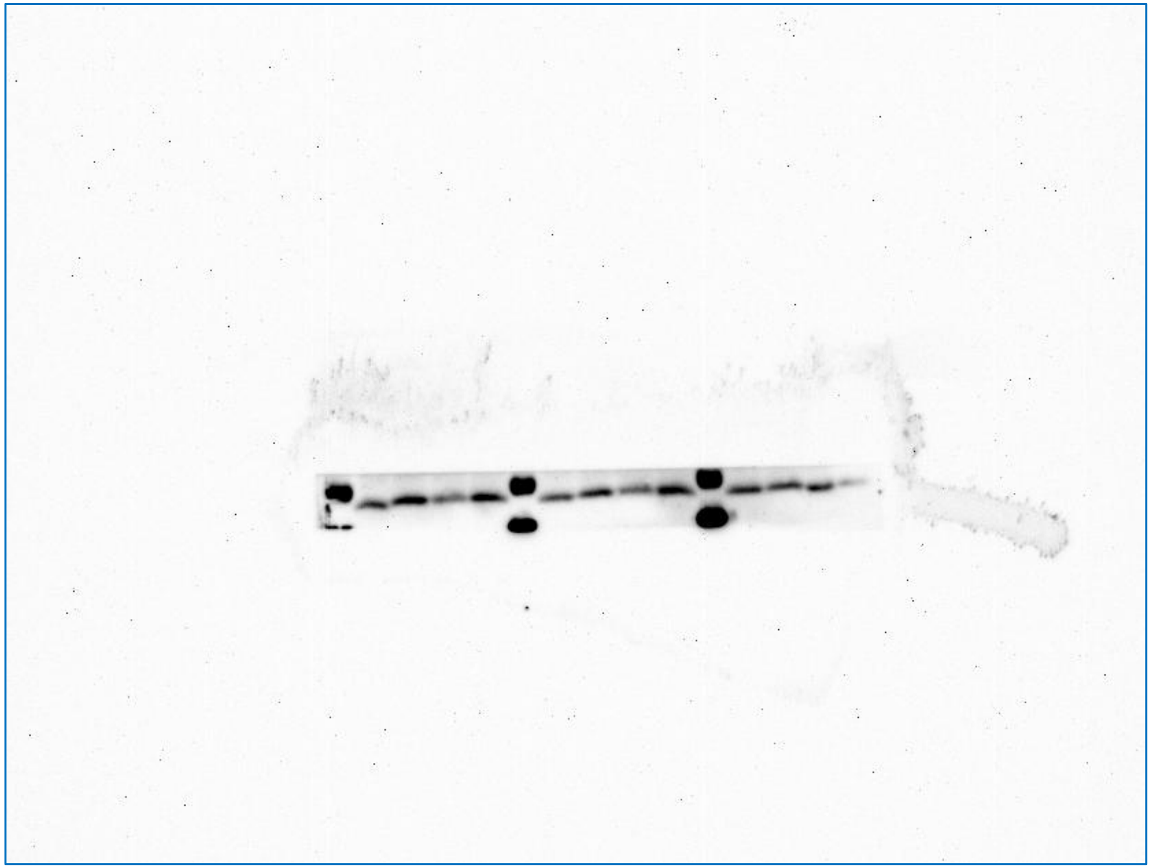

Figure 4 LIAS

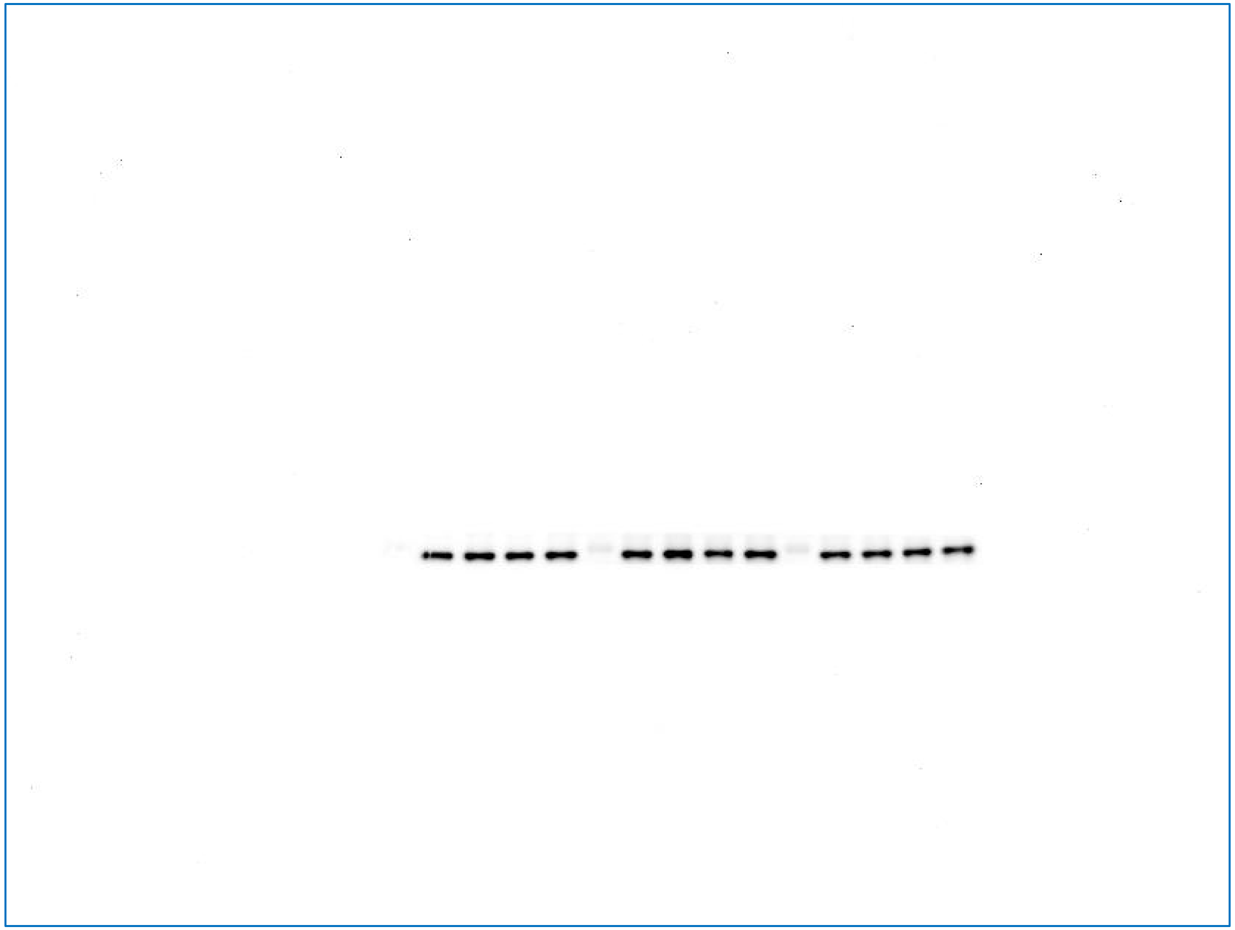

Figure 4 ATP7B

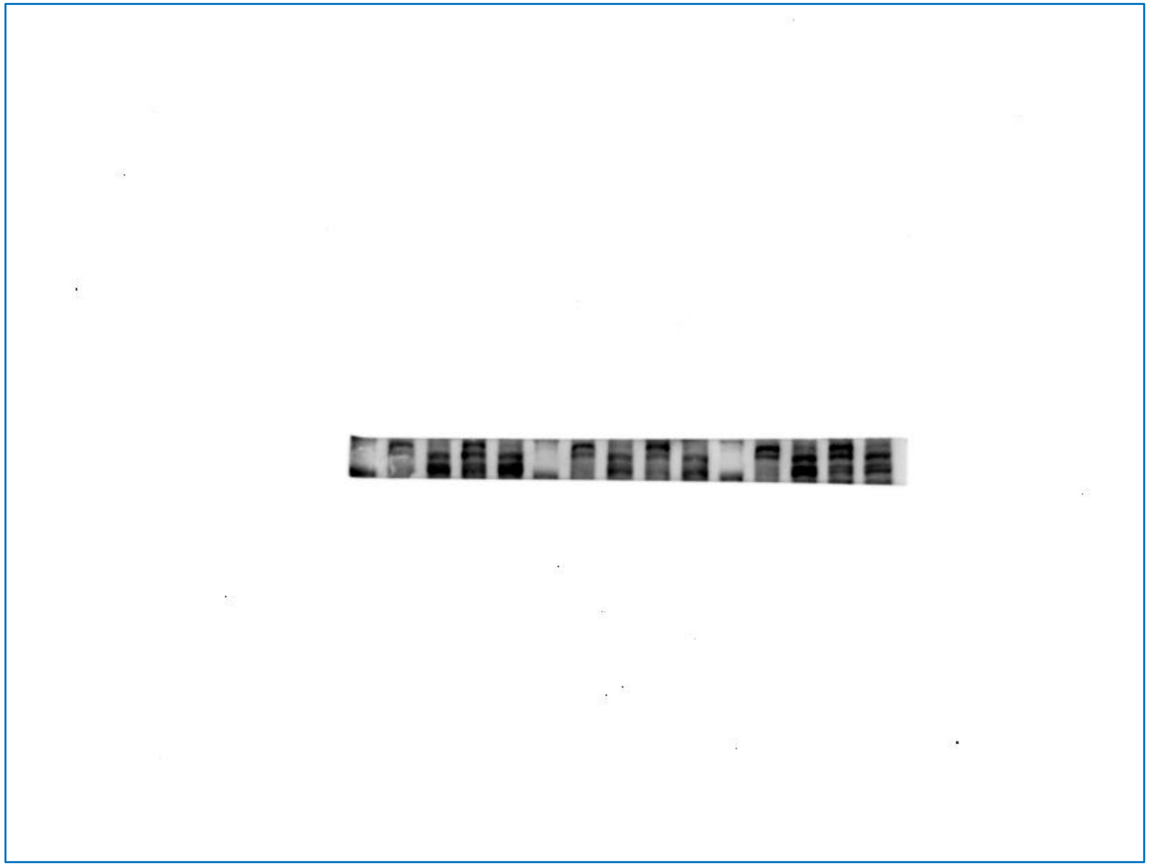

Figure 4 Lip-DLAT

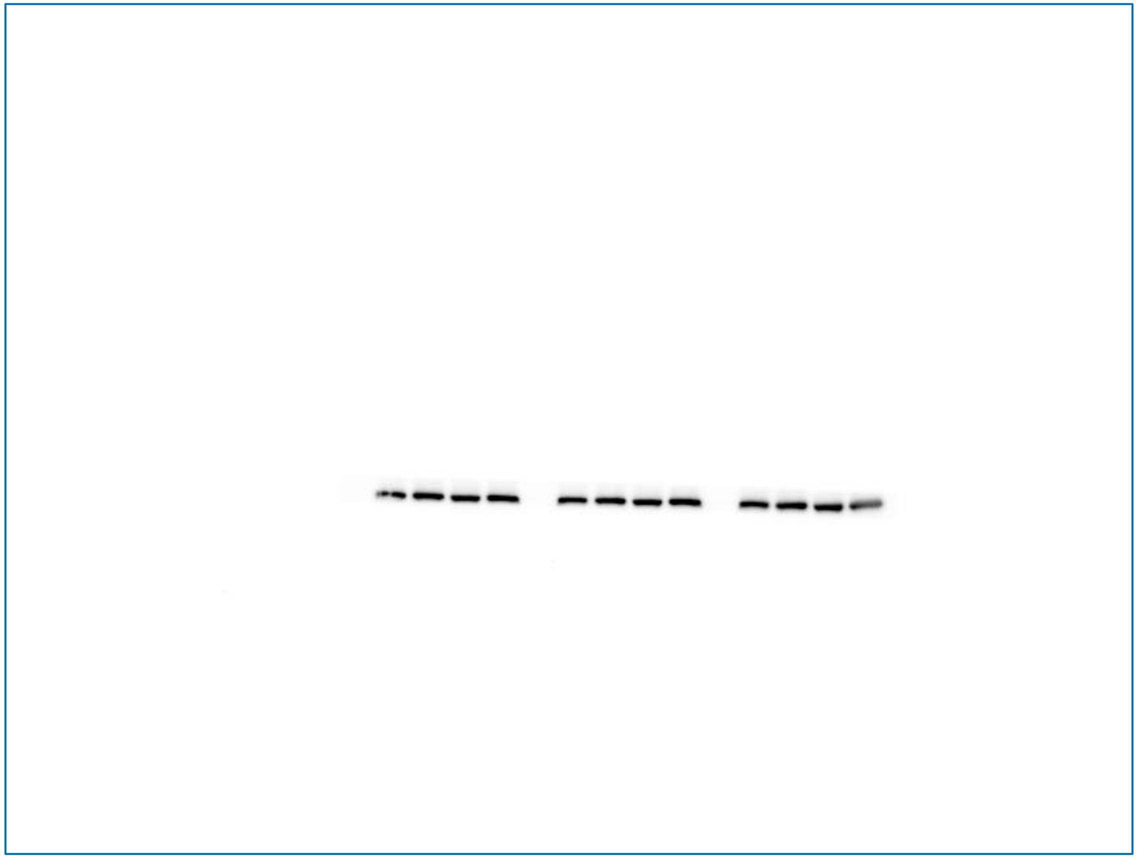

Figure 4 GPADH

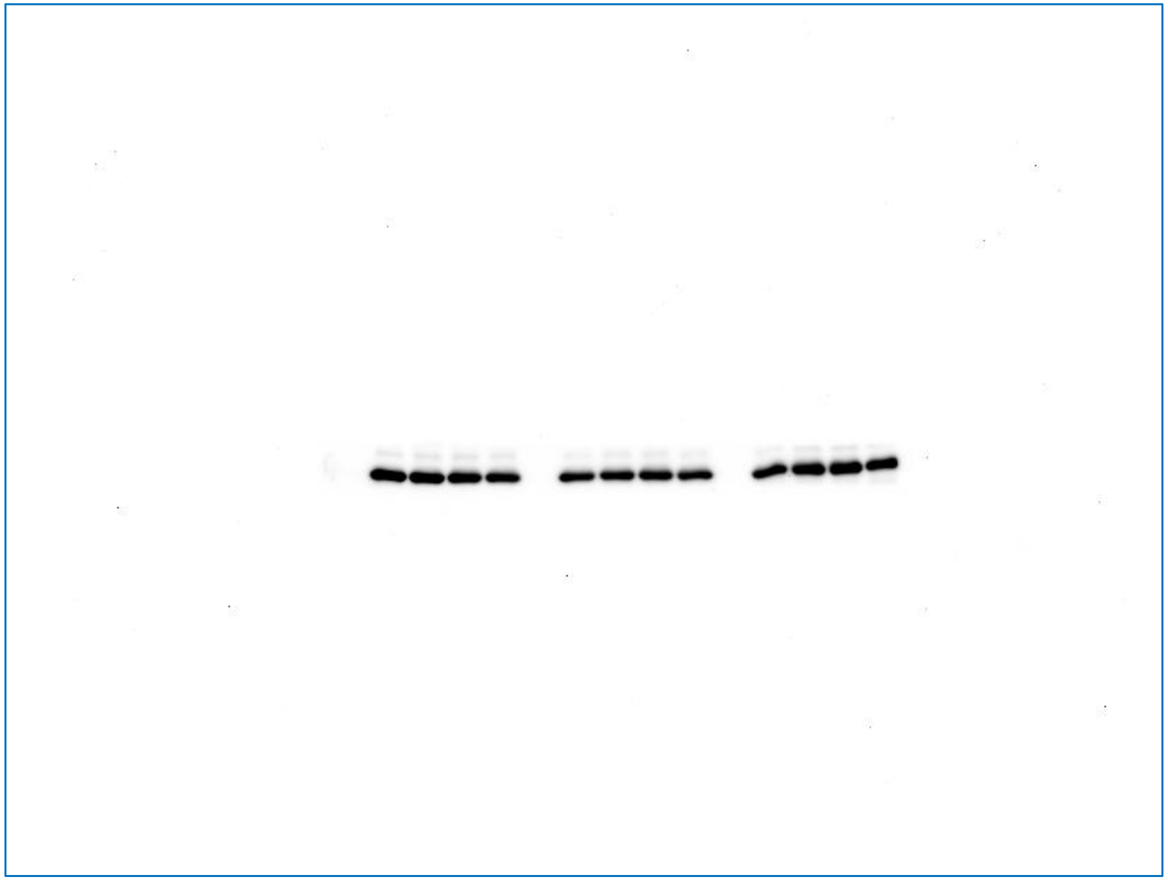

Figure 4 DLST

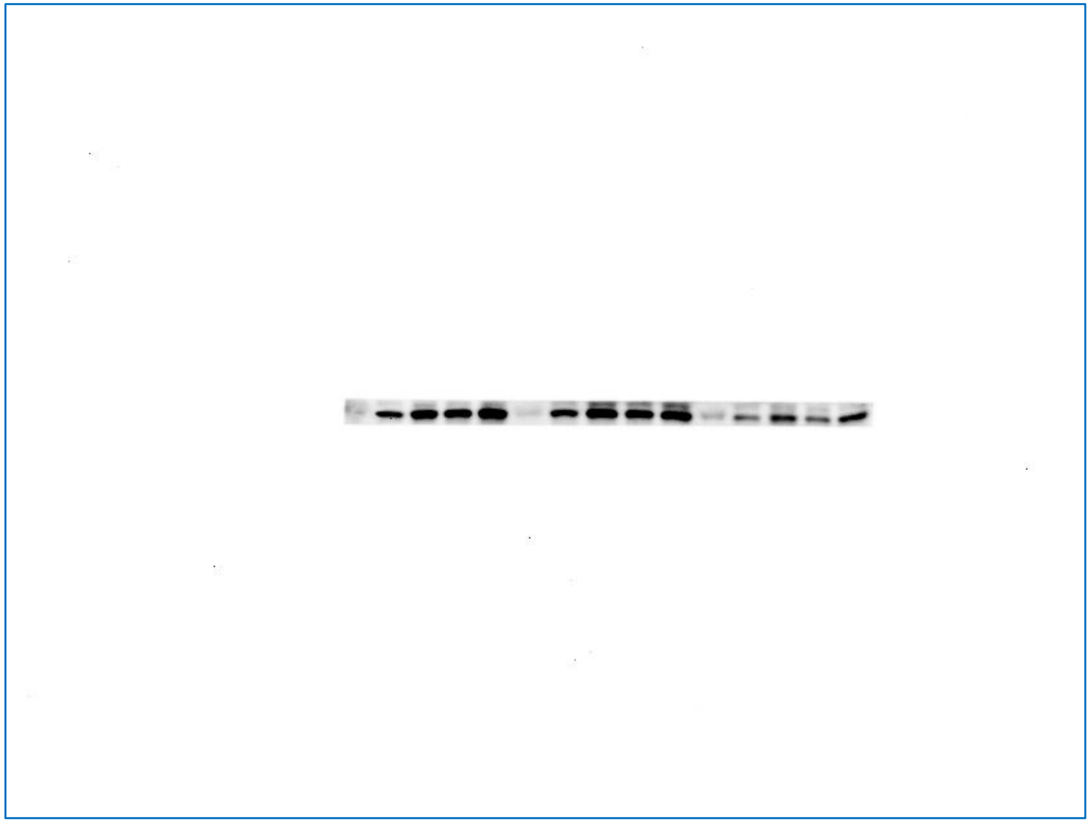

Figure 4 PDHB

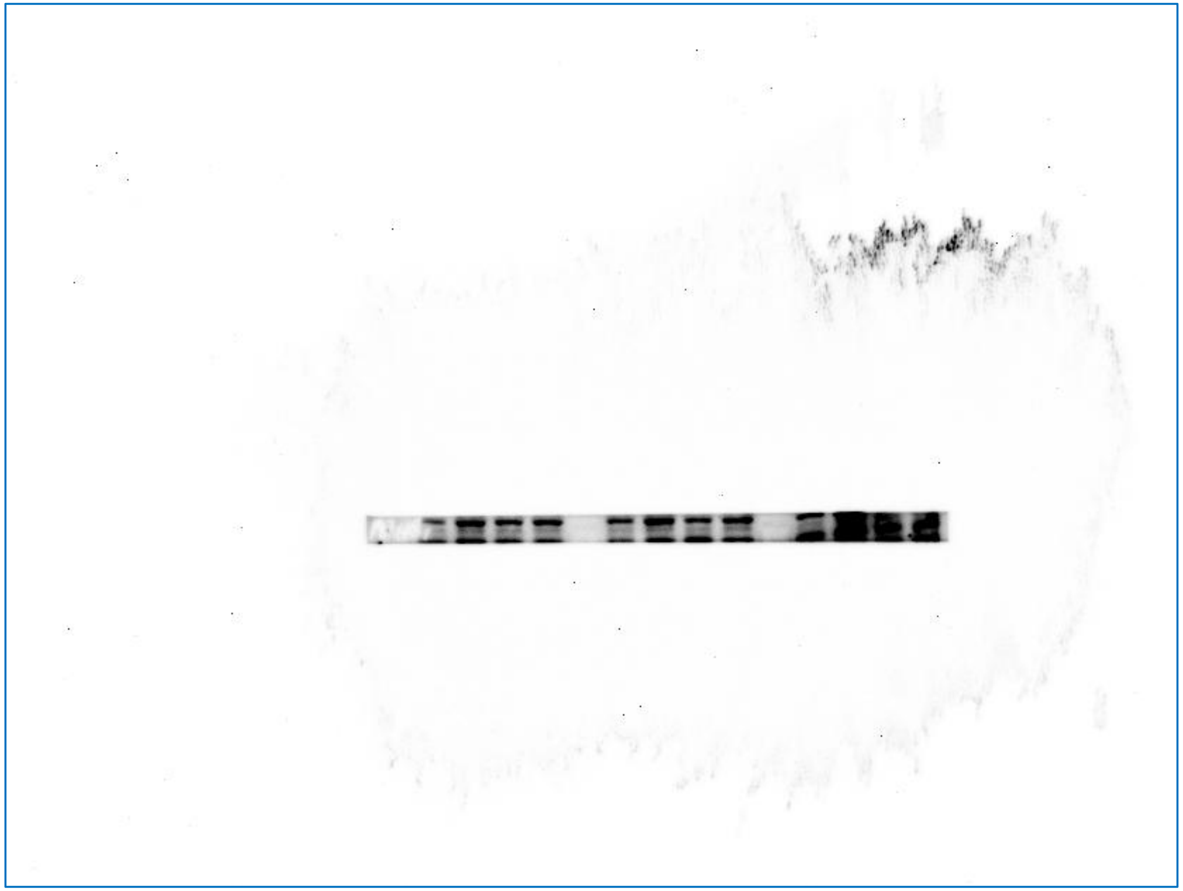

Figure 4 SDHB

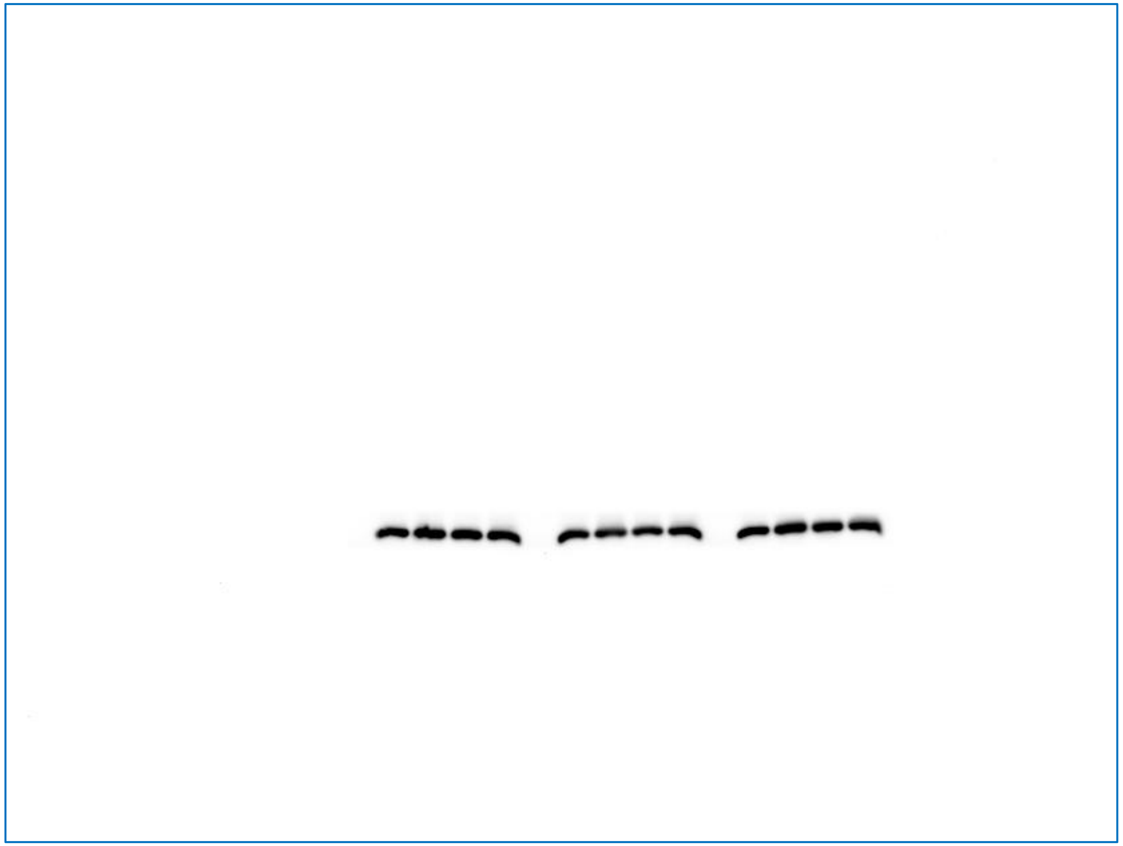

Figure 4 HSP70

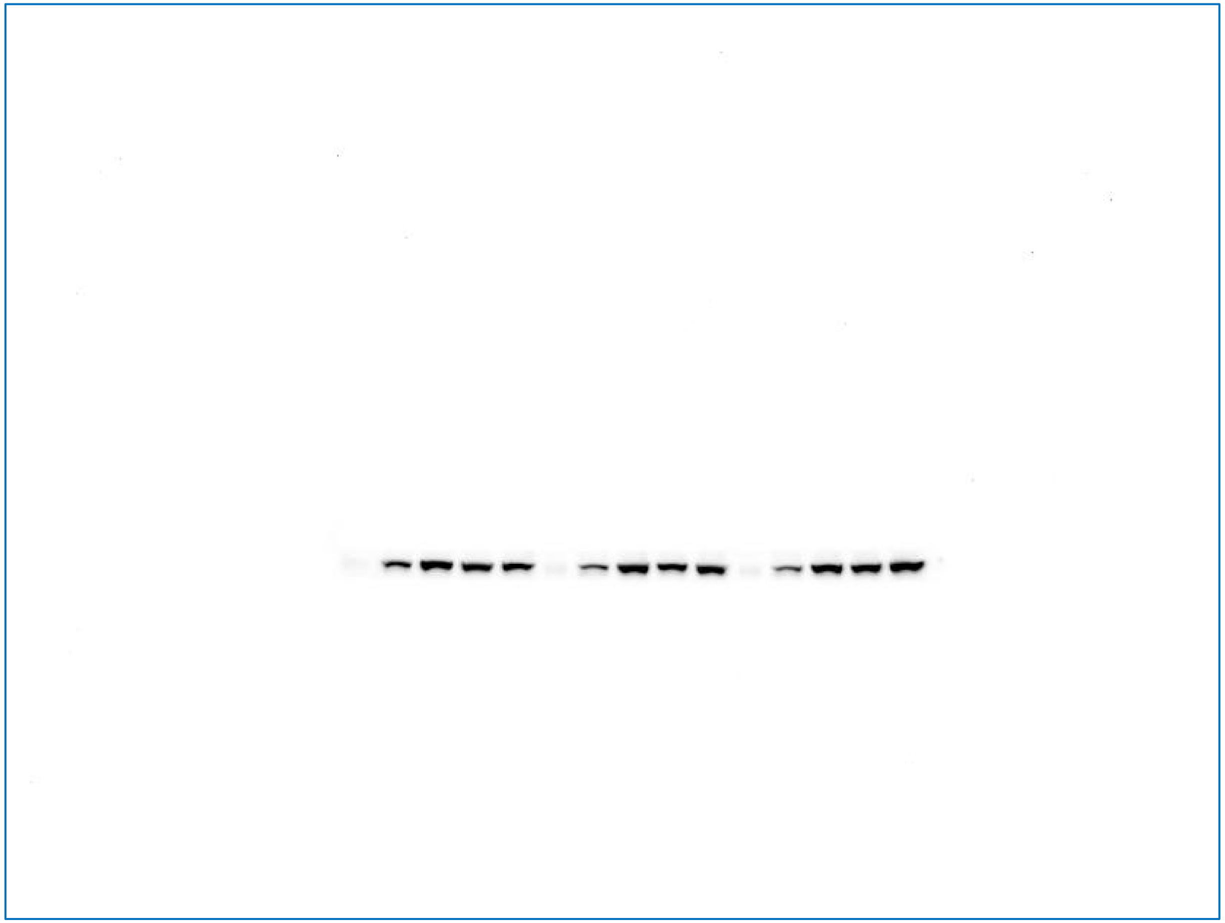

Figure 4 GPADH

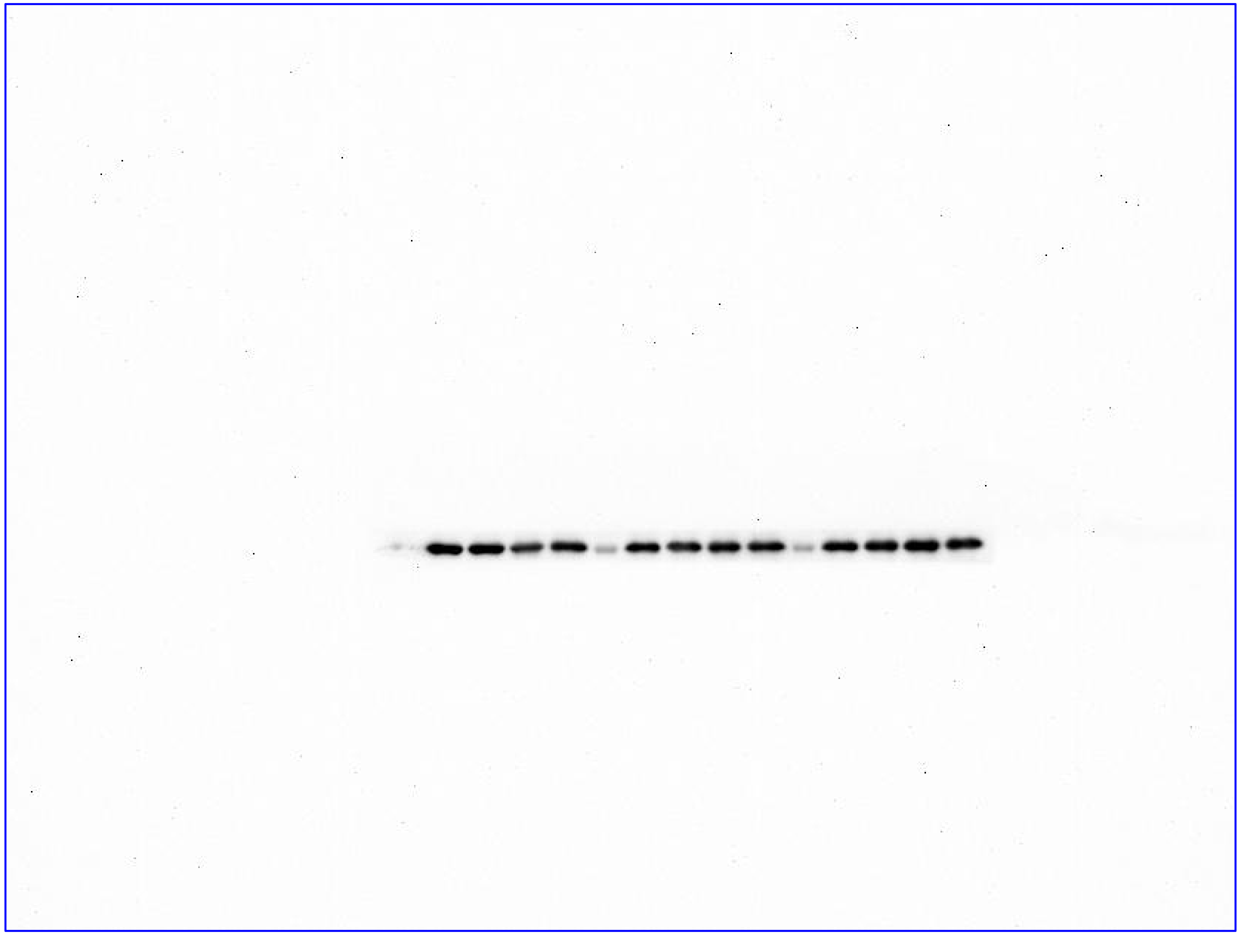

Figure 6 TLR4

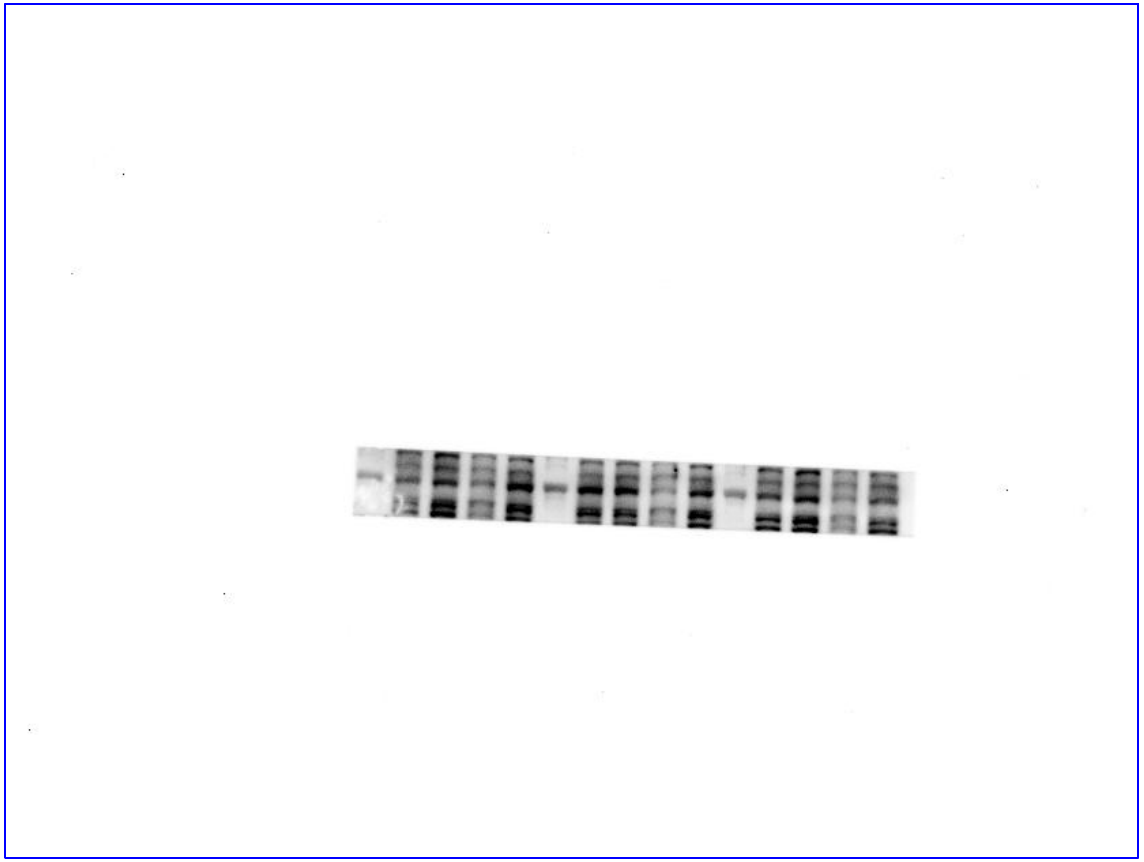

Figure 6 NF-κB

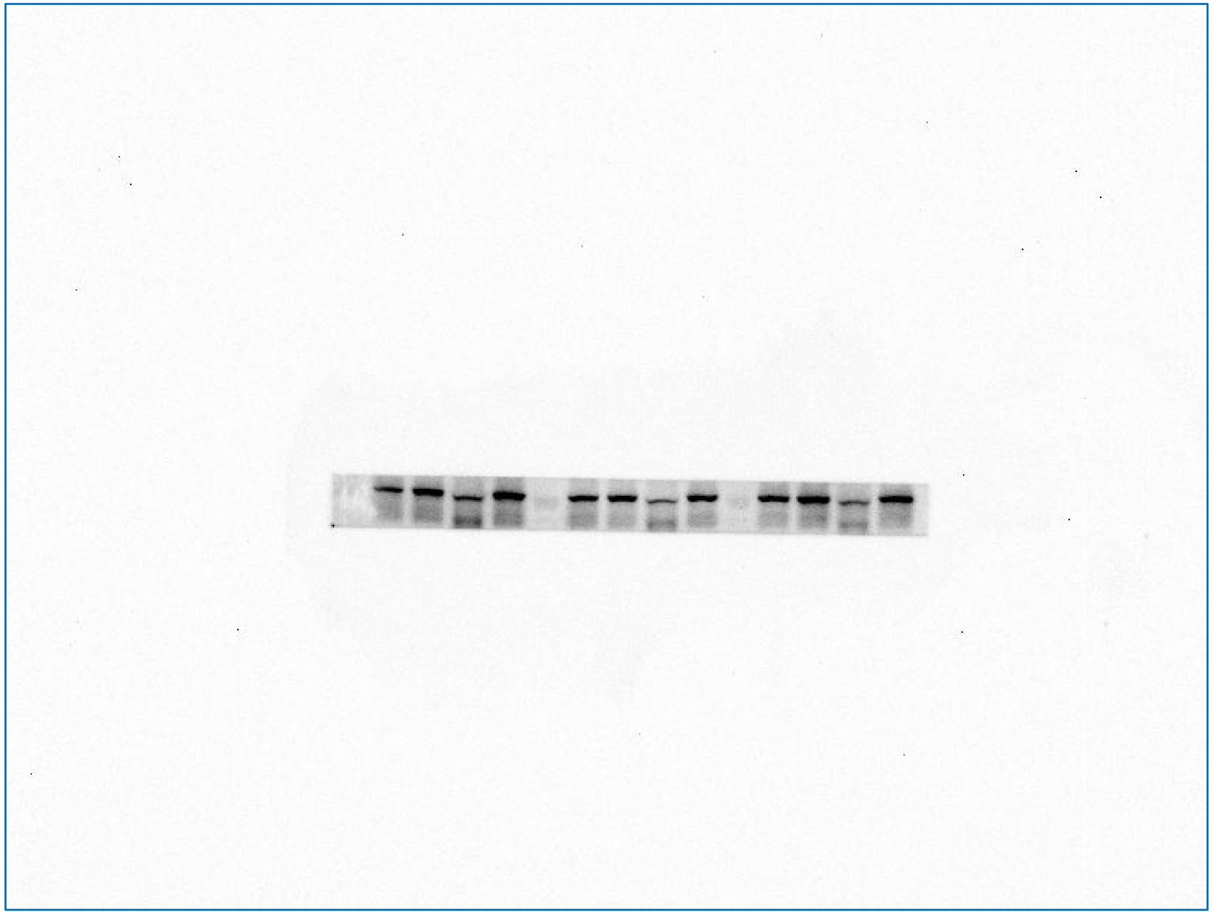

Figure 6 TNF-α

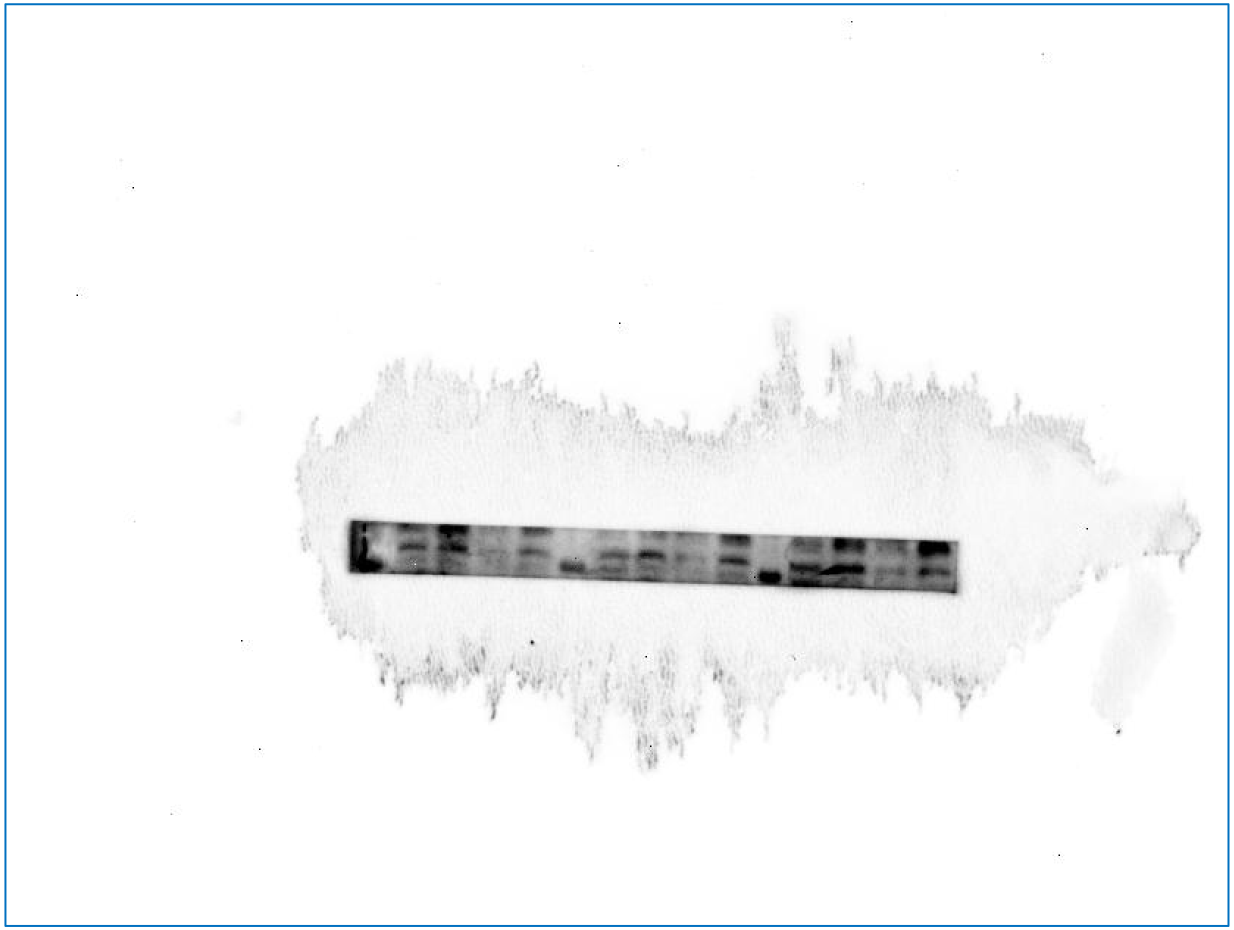

Figure 6 NLRP3

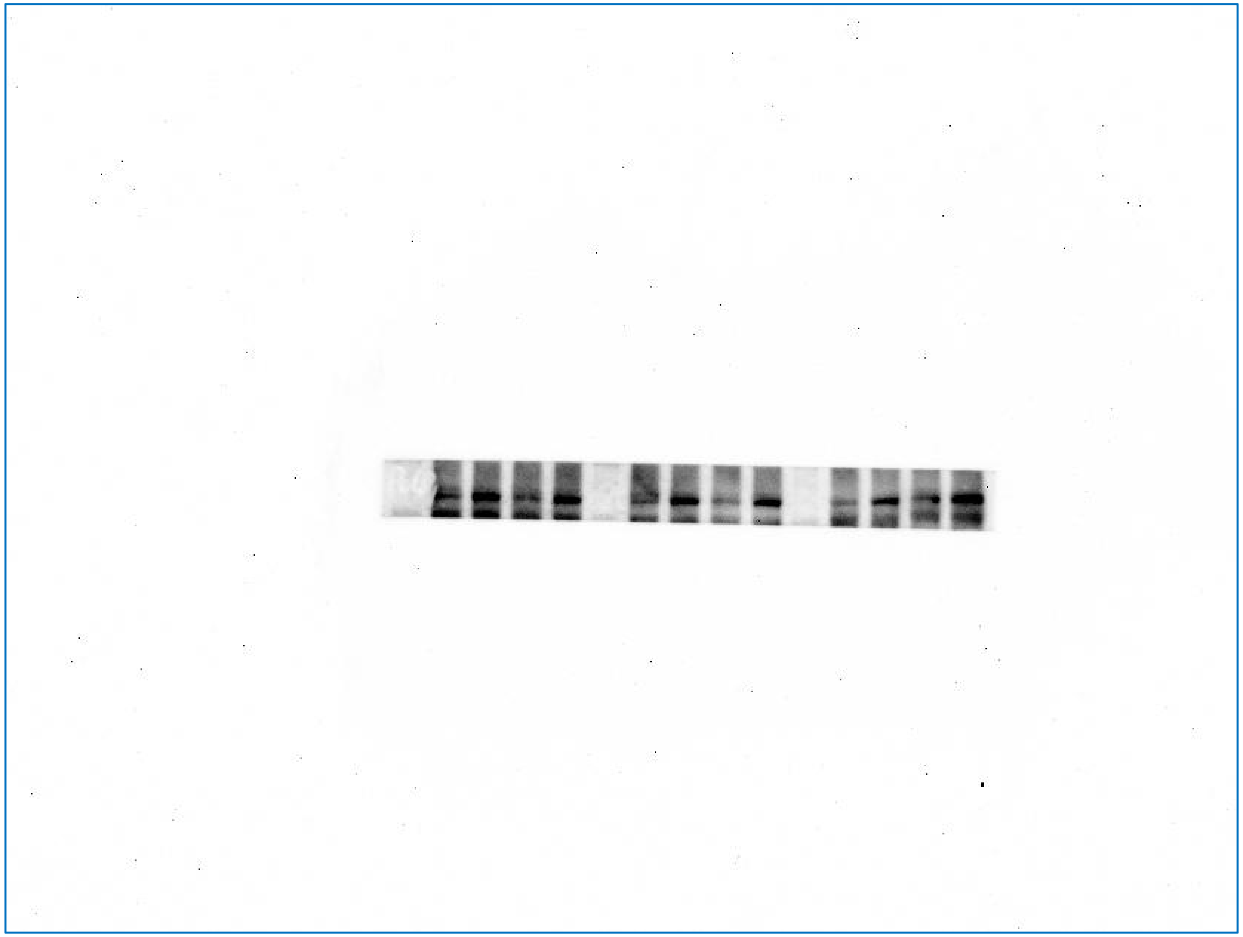

Figure 6 ASC

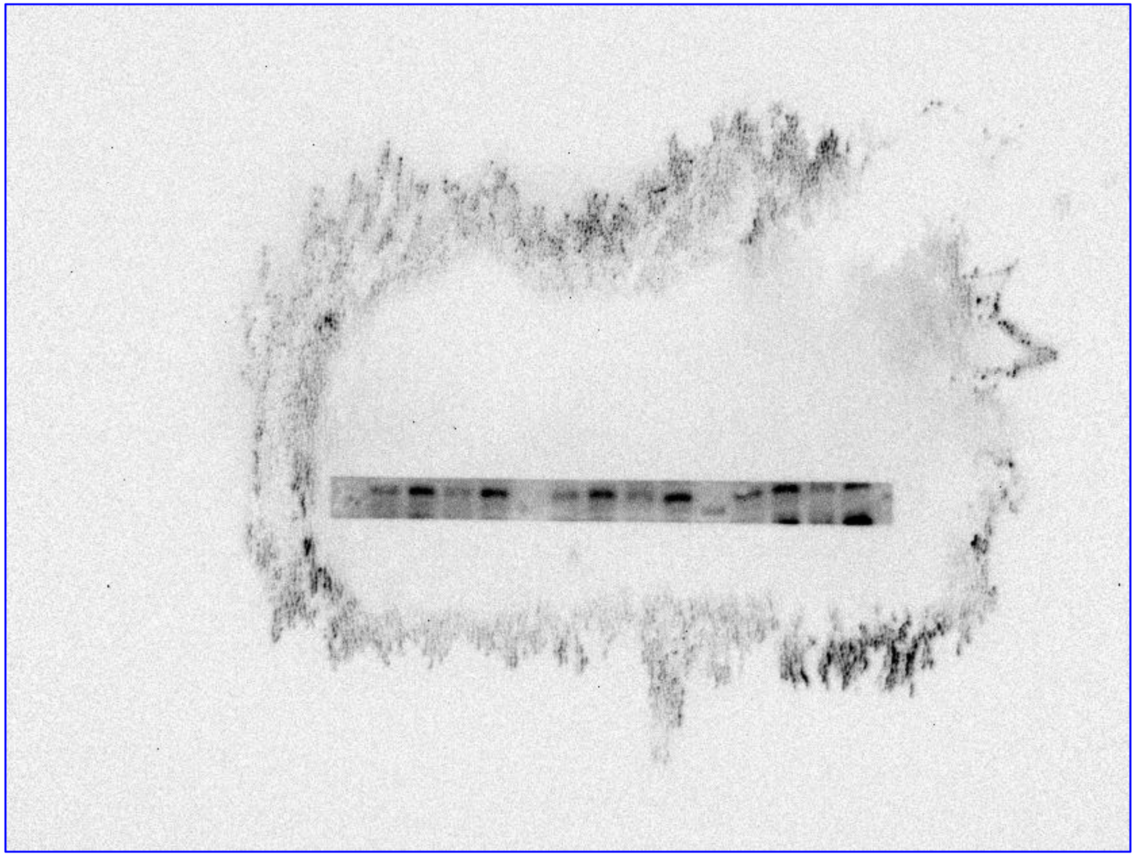

Figure 6 Pro-Caspase1

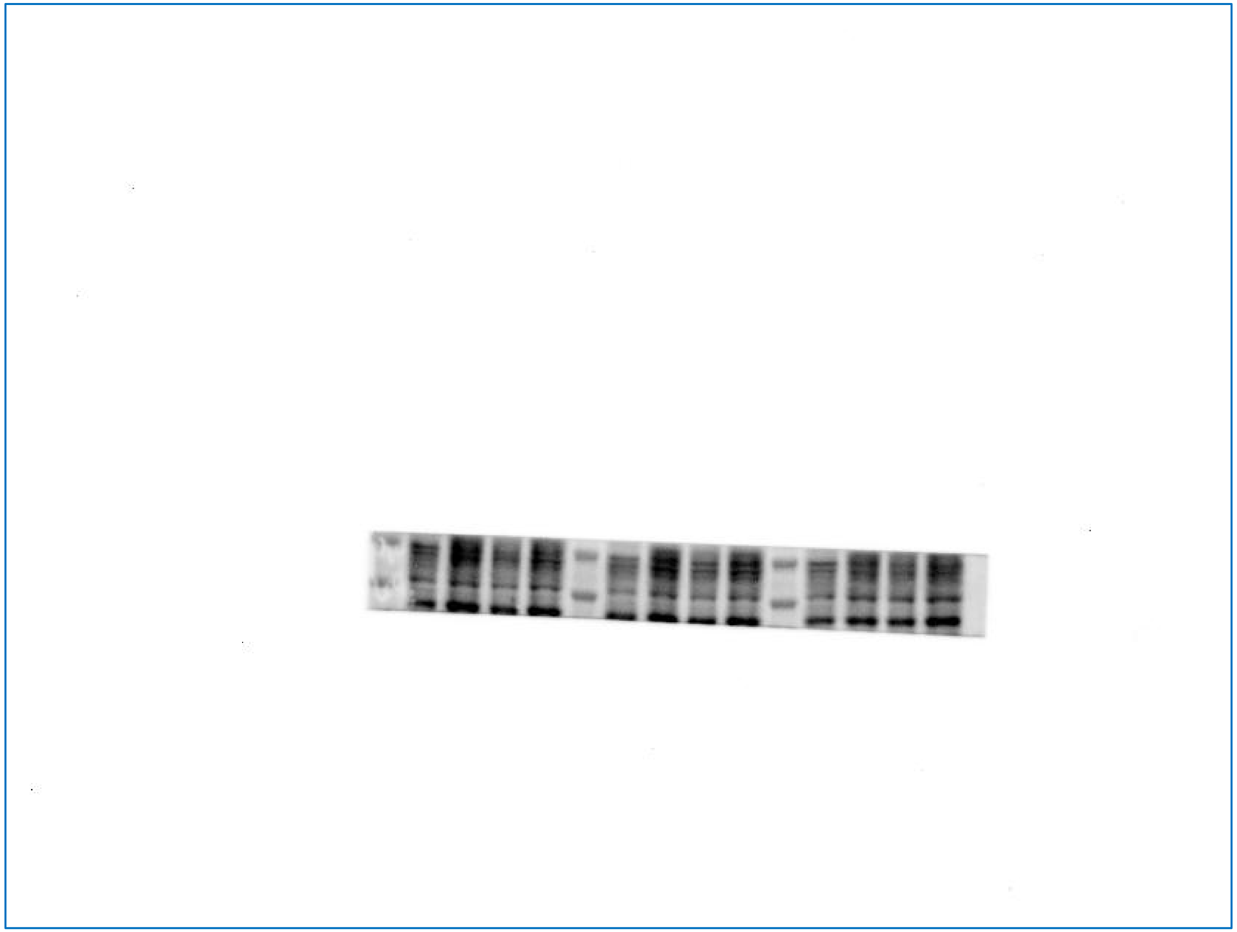

Figure 6 Cleaved-Caspase1

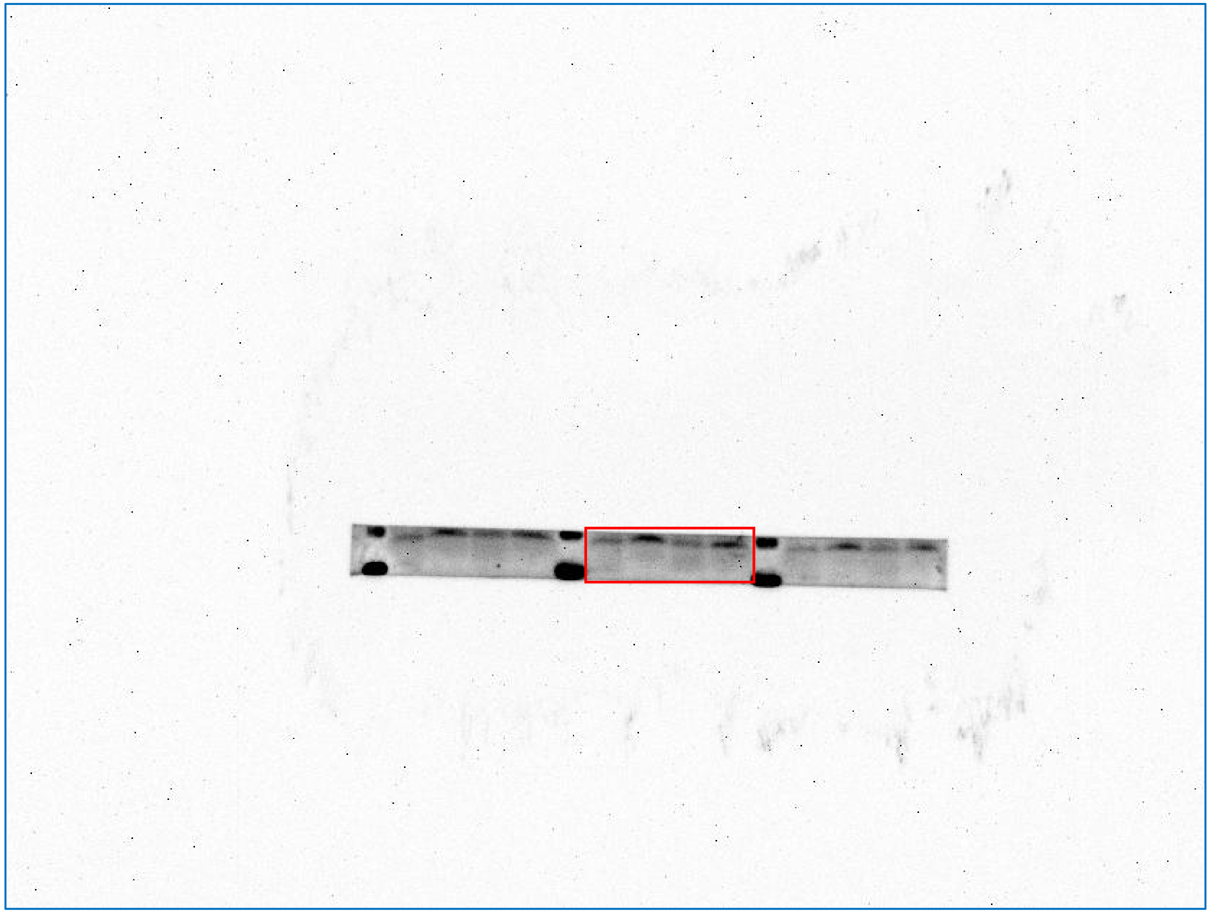

Figure 6 GSDMD-N

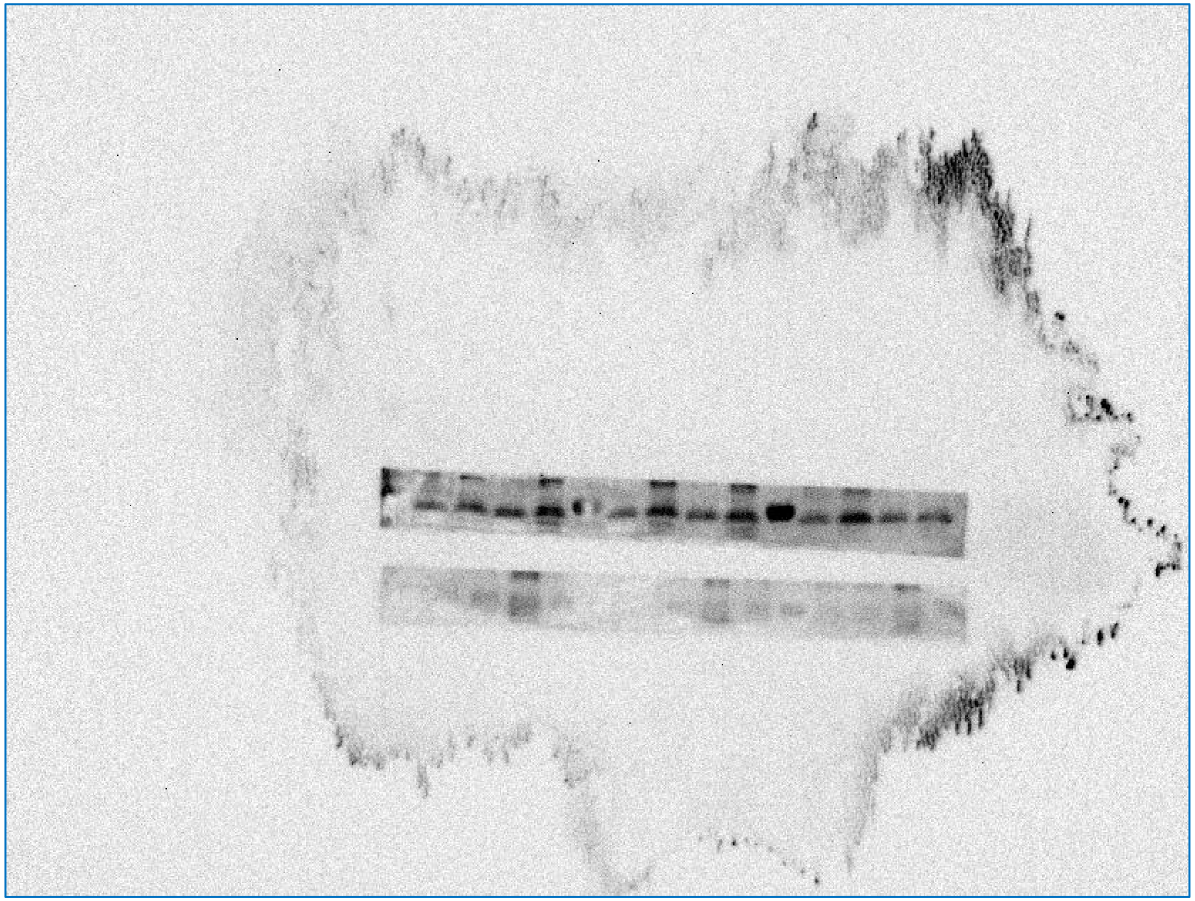

Figure 6 IL-18

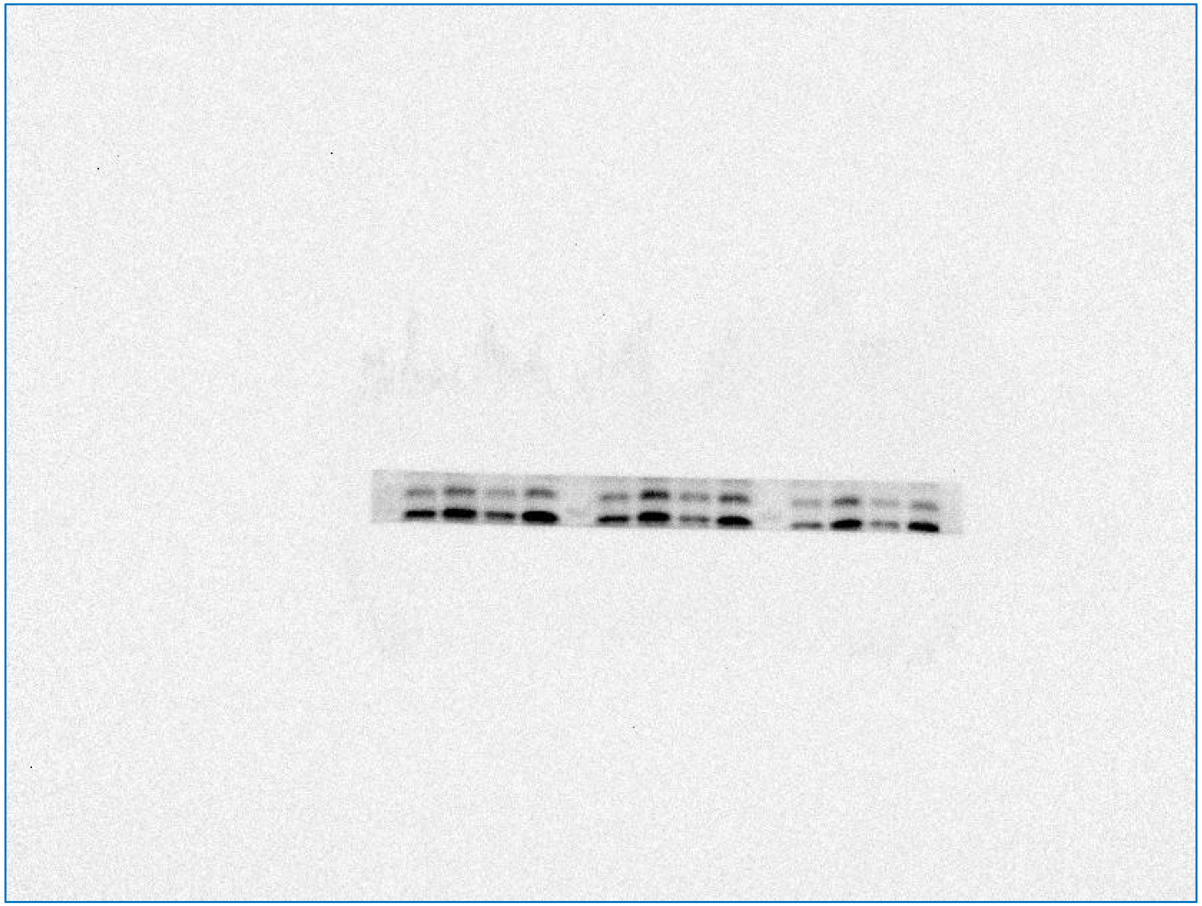

Figure 6 IL-1β

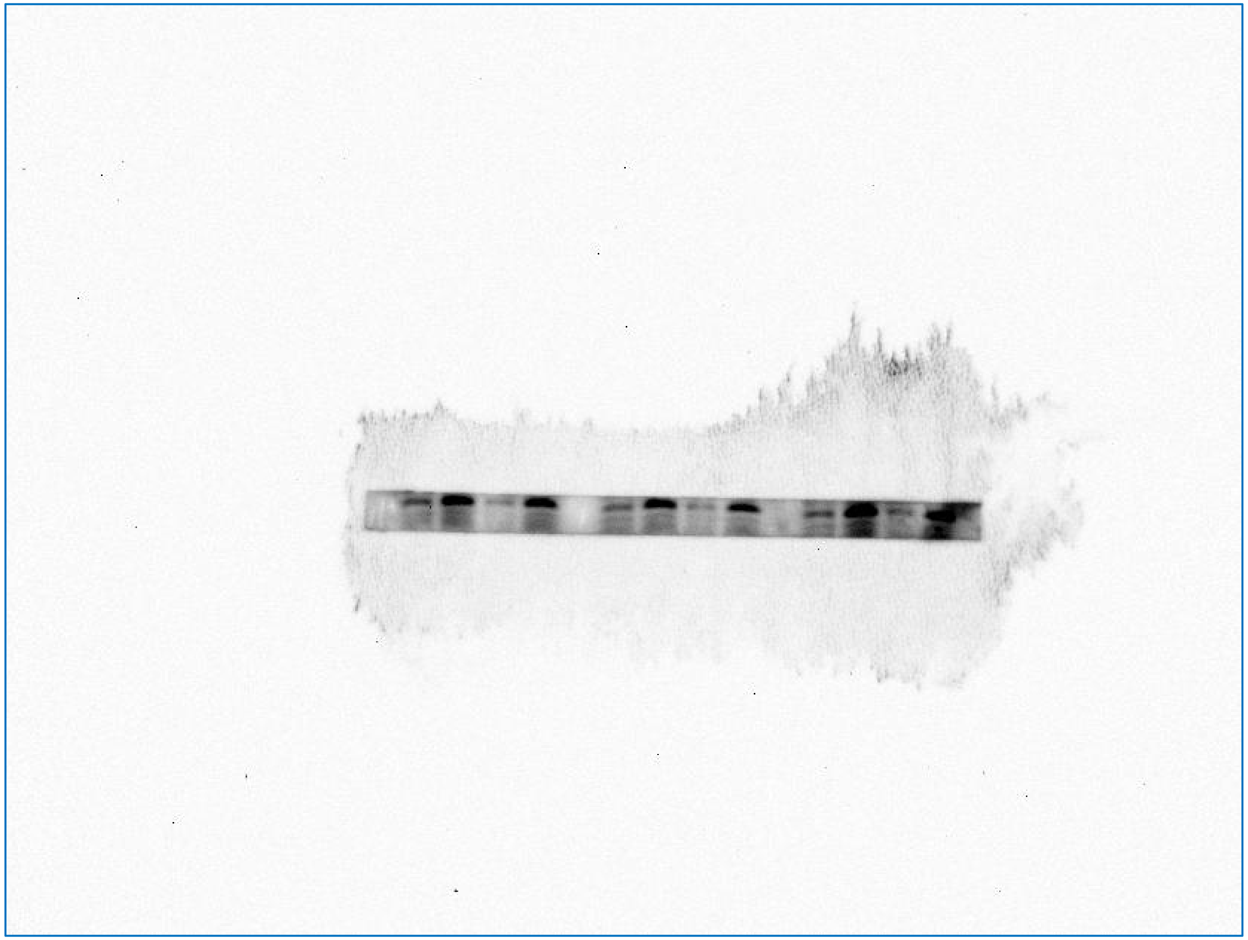

Figure 6 GPADH

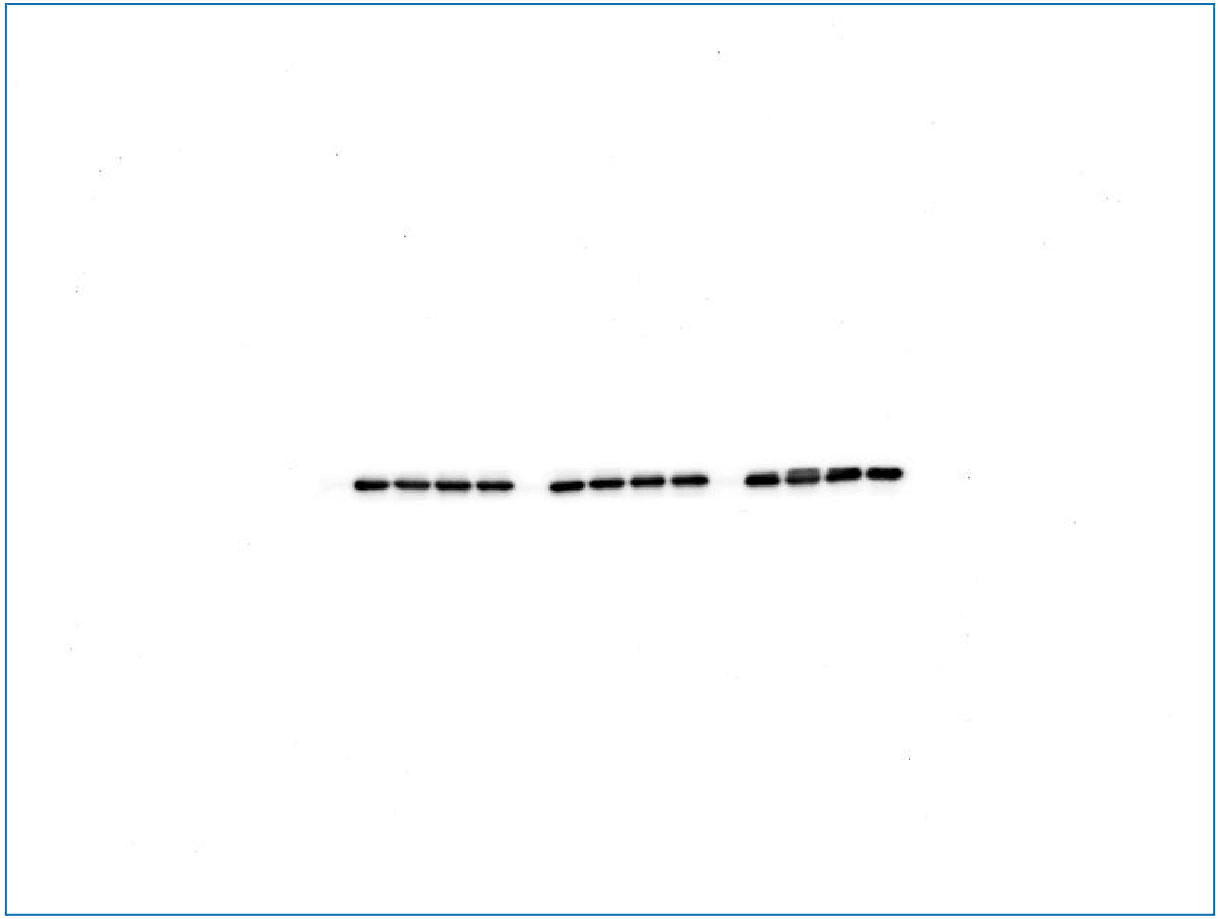

Figure 6 IL-17

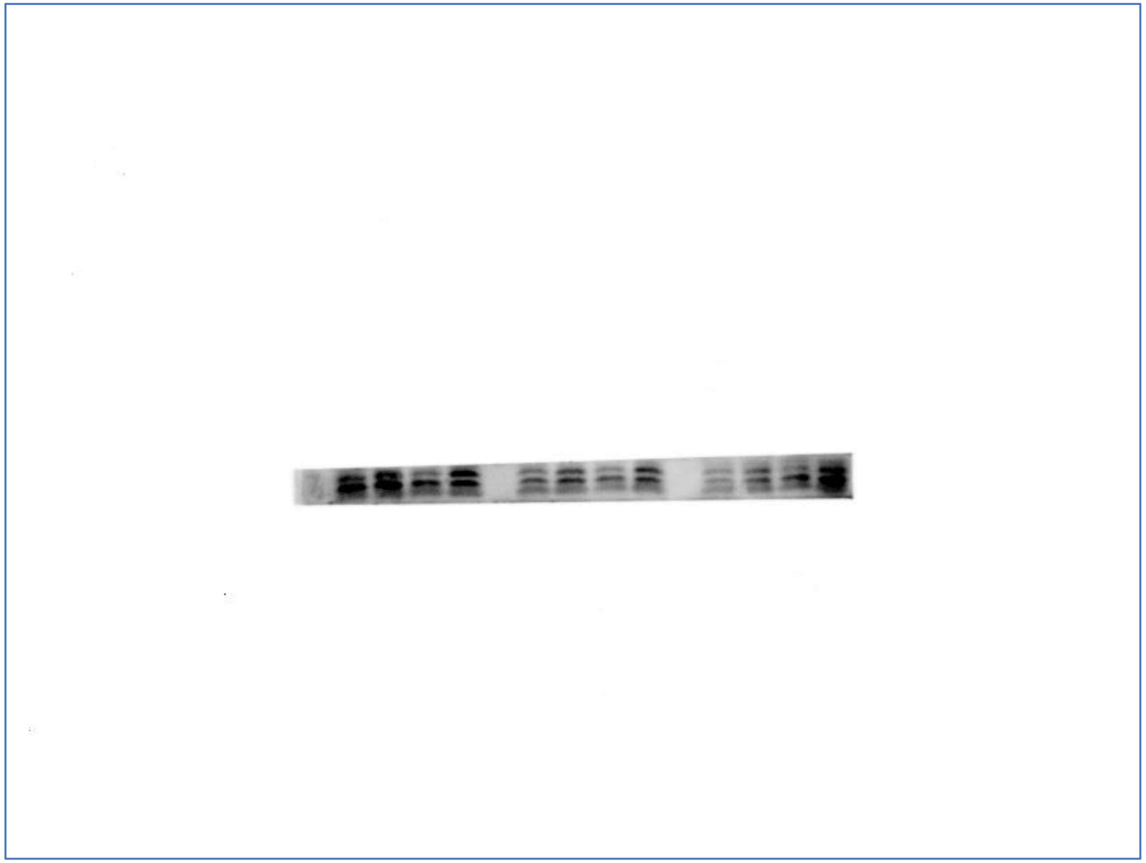

Figure 6 GPADH

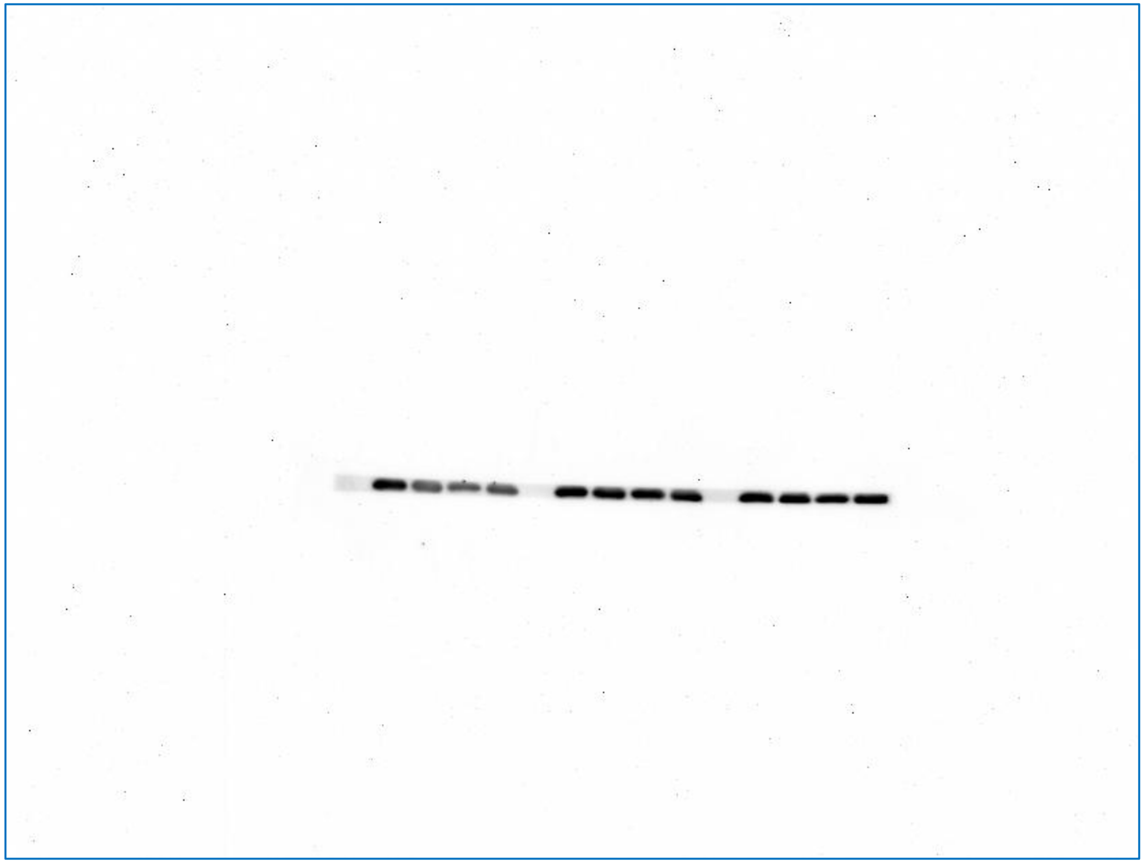

Supplement: Supplementary file 1 — Supplementary Information. [file 41598_2024_64981_MOESM1_ESM.pdf]
